# Dual inhibitors of hepatitis C virus and hepatocellular carcinoma: design, synthesis and docking studies

**DOI:** 10.4155/fsoa-2017-0075

**Published:** 2017-10-25

**Authors:** Mostafa MM El-Miligy, Samia M Rida, Fawzia A Ashour, Mona H Badr, Ehab M El-Bassiony, Maha A El-Demellawy, Ashraf M Omar

**Affiliations:** 1 Pharmaceutical Chemistry Department, Faculty of Pharmacy, Alexandria University, Alexandria 21521, Egypt; 2 Medical Biotechnology Department, Genetic Engineering & Biotechnology Research Institute, City for Scientific Research & Technology Applications, Alexandria, Egypt

**Keywords:** benzimidazole, benzofuran, docking, HCC inhibitors, HCV inhibitors, thiazolidinone

## Abstract

**Aim:**

Simultaneous inhibition of hepatitis C virus (HCV) and hepatocellular carcinoma (HCC) may enhance anti-HCV effects and reduce resistance and side effects.

**Results/methodology:**

Novel hybrid derivatives were designed and synthesized to exhibit dual activity against HCV and its associated major complication, HCC. The synthesized compounds were screened for their potential activity against HCV and HCC. Compounds **5f, 5j, 5l, 5p, 5q, 5r, 6c** and **6d** exhibited potential *in vitro* anticancer activity against HCC cell line HepG2, while compounds **5a, 5l, 5p** and **5v** showed *in vitro* anti-HCV activity. Docking studies suggested that the newly synthesized compounds could suppress HCC through VEGFR2 tyrosine kinase inhibition.

**Conclusion:**

Compounds **5l** and **5p** exhibited dual activity against HCV and HCC *in vitro*.

## Lay abstract

There is a close relation between chronic hepatitis C virus (HCV) infection and development of hepatocellular carcinoma (HCC). Dual inhibition of HCV and HCC could enhance anti-HCV activity and reduce resistance of HCC to treatment. Novel compounds were designed and synthesized to exhibit dual activity against HCV and HCC. The synthesized compounds were screened for their potential *in vitro* activity against HCV and the HCC HepG2 cell line. Compounds **5l** and **5p** exhibited dual activity against HCV and HCC *in vitro*. Further studies predicted that the target compounds could suppress HCC.

## Graphical abstract

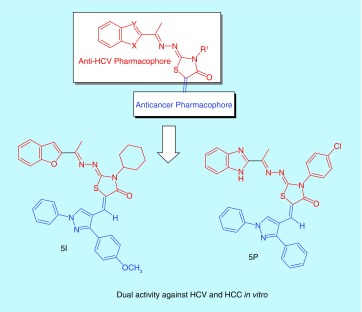

Hepatitis C virus (HCV) is a contagious liver disease. Chronic HCV infection results in chronic inflammation that can lead to liver fibrosis, cirrhosis, hepatocellular carcinoma (HCC) and death [1]. About 71 million people are chronically infected and at risk of developing liver cirrhosis and/or HCC. Approximately 399,000 people die from hepatitis C-related liver diseases every year [2].

The standard treatment therapy for patients with chronic HCV infection consists of a combination of pegylated IFN-α injections and oral ribavirin for 24–48 weeks. However, this therapy suffers from many limitations including limited efficacy against some virus genotypes and severe side effects such as anemia.

The discovery of direct-acting antiviral agents led to development in the treatment of chronic HCV infection. The preferred treatment regimen depends on the viral genotype, if the patient has cirrhosis or not, on previous drug treatment and drug costs [3]. In 2011, the US FDA approved the new antiviral drugs boceprevir and telaprevir to be used with the standard therapy [4]. This new combination increased the incidence of anemia [4]. In 2013, the FDA approved simeprevir and sofosbuvir to be used in combination with interferon and ribavirin. However, this combination showed excellent activity only against some virus genotypes [4].

Worldwide, HCC is one of the top three cancer killers [5]. In 2012, 782,200 cases were diagnosed and were responsible for 746,000 deaths [6]. Chronic HCV increases risk of developing HCC 20–30-fold as compared with uninfected patients. Nearly 2.5% of chronic HCV patients develop HCC [7]. Many reports have illustrated the role of some viral proteins, particularly phosphorylated NS5A, in the development of HCC. The HCV protein NS5A is activated by human tyrosine kinase to produce the active phosphorylated form [8], which upregulates COX-2 expression and promotes the release of matrix metalloprotinase-2 and 9 associated with tumor progression and recurrence in HCC patients [9,10]. In addition, phosphorylated NS5A interacts with and activates certain kinases involved in signal transduction pathways responsible for proliferation of HCC cells [10]. Moreover, phosphorylated NS5A inhibits apoptosis through inhibiting p53 function and inactivating the cell defense mechanism RNA-dependent protein kinase [10–13]. Also, the highly vascular nature of HCC indicates the activation of angiogenic signaling pathways, activated mainly through receptor tyrosine kinases [14].

Currently, there is no effective therapy for HCC. Sorafenib is approved as first-line treatment but it has severe adverse side effects such as dermatological adverse effects [15], diarrhea [16] or arterial hypertension [17]. Regorafenib has recently been approved as second-line therapy for HCC after failure of sorafenib [18,19]. The search for more effective molecular agents than sorafenib or combinations of therapy might improve response and survival rates in HCC patients. Radioembolization [20] and immunotherapy [21] are still in clinical trials. Thus, there is an urgent need for new therapeutic agents for HCV infection and its associated major complication, HCC. A literature survey revealed that many derivatives containing benzofuran, its bioisostere benzimidazole or thiazolidinone **I**-**VI** were active against HCV (Figure 1) [22–27]. On the other hand, many compounds containing (2-oxoindolin-3-ylidene) moiety such as sunitinib, SU6668, NP603, NP506 and BIBF 1120 (**VII-XI**) showed anticancer activity against many tumors through inhibition of kinases (Figure 2) [14,28–31]. In addition, 1,3-diaryl-1*H*-pyrazole moiety is an interesting scaffold for the design of kinase inhibitors. Many compounds containing this moiety (**XII-XIV**) displayed kinase inhibitory activity and showed antiproliferative activity against different types of cancer [32–35]. It was found that 2-oxoindolin-3-ylidene scaffold in the multikinase inhibitor sunitinib is responsible for hydrogen bond interaction with the hinge region [36] while the pyrazole ring was reported to recognize the ATP-binding site [36] either by forming hydrogen bonds [37], arene–cation interactions or by contribution to the hydrophobic interactions [33,38]. Therefore, we can expect that the attachment of well-known kinase inhibitor pharmacophores such as substituted 2-oxoindolin-3-ylidene or 3-aryl-1-phenyl-1*H*-pyrazole will enhance the kinase inhibitory and anticancer activity of our compounds to be active against HCC.

**Figure 1.**
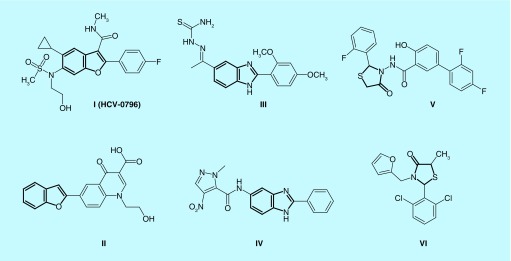
**Benzofuran, benzimidazole or thiazolidinone derivatives active against the hepatitis C virus.**

**Figure 2.**
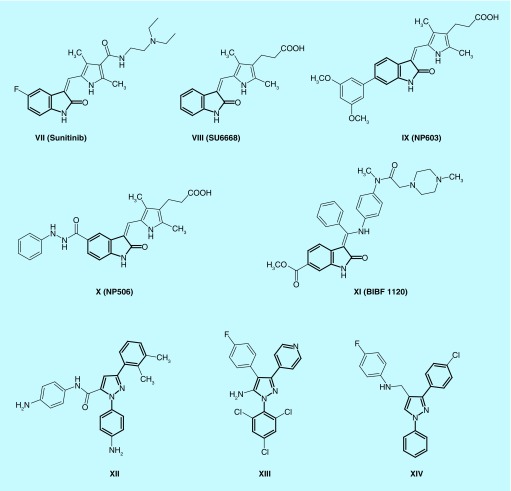
**Some anticancer kinase inhibitors containing 2-oxoindolin-3-ylidene or 1,3-diaryl-1H-pyrazole moieties.**

Consequently, we focused on the design and synthesis of novel hybrid compounds as dual anti-HCV and anti-HCC. The target compounds were designed to hybridize an anticancer kinase inhibitor pharmacophore composed of 2-oxoindolin-3-ylidene or 1,3-diaryl-1*H*-pyrazole to an anti-HCV pharmacophore composed of benzofuran or its bioisostere benzimidazole linked to thiazolidinone ring at position 2 through three atoms spacer. The substituents at position 3 of thiazolidinone moiety were chosen to include either aliphatic cyclohexyl group or aromatic unsubstituted phenyl or substituted with electron withdrawing (Cl) or donating (OCH_3_) groups. Besides, the phenyl moiety at position 3 of 1,3-diaryl-1*H*-pyrazole pharmacophore was substituted with either electron withdrawing (Cl) or donating (OCH_3_) groups to change π-electron density of the target compounds and hence binding affinity and activity (Figures 3 & 4).

**Figure 3.**
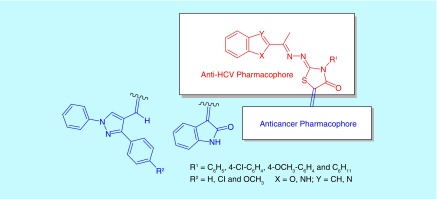
**Design of dual anti-hepatitis C virus and anticancer hybrids.**

**Figure 4.**
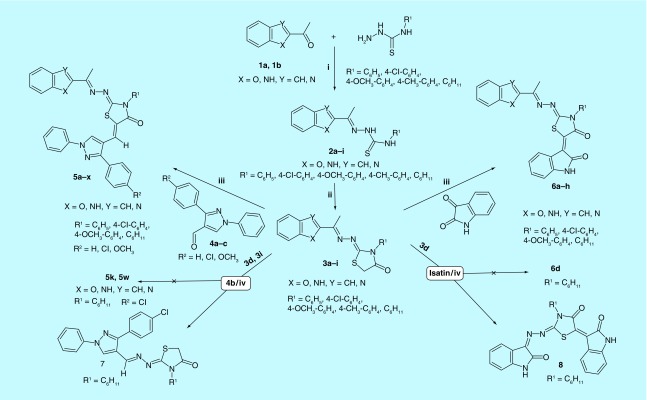
**Synthesis of the target dual anti-hepatitis C virus and anticancer hybrids (5a-x) and (6a-h).** Reagents: **(i)** Ethanol; **(ii)** ClCH2COOH, CH3COOH, CH3COONa; **(iii**) Piperdine, Dry Dioxane; **(iv)** CH3COOH, CH3COONa.

## Results & discussion

### Chemistry

The target compounds were prepared (Figure 4) through the reaction of 2-acetylbenzofuran or 2-acetyl benzimidazole with substituted thiosemicarbazides in ethanol containing few drops of glacial acetic acid as a catalyst [39,40] to yield the corresponding thiosemicarbazones, which were then cyclized using chloroacetic acid and anhydrous sodium acetate in glacial acetic acid [39,41] to give the key intermediates, 4-thiazolidinones. Infrared (IR) spectra of thiosemicarbazones intermediates (**2a-i**) were characterized by the disappearance of absorption band due to C = O at 1678–1674 cm^-1^ and the appearance of N-C = S amide I, II, III and IV bands at 1571-1560, 1304-1300, 1187-1180, 927-921. Their ^1^H-NMR spectra showed a singlet at 2.34–2.53 p.p.m. corresponding to -N = C-CH_3_ in addition to two deuterium exchangeable singlets of H-N-(C = S)-N-H at 7.99–10.43 and 10.43–11.02 p.p.m. The other signals appeared at their expected chemical shifts. IR spectra of 4-thiazolidinone intermediates (**3a-i**) were characterized by the disappearance of N-C = S amide I, II, III and IV bands and appearance of C = O band at 1726–1714 cm^-1^. Their ^1^H-NMR spectra were characterized by the disappearance of H-N-(C = S)-N-H signals at 7.99–11.02 p.p.m. and the appearance of a singlet at 3.94–4.17 p.p.m. attributed to thiazolidinone C^5^-H_2_. The other protons appeared at their expected chemical shifts. 4-thiazolidinones were subjected to Knoevenagel condensation with either 3-aryl-1-phenyl-pyrazole-4-carbaldehydes (**4a-c**) or isatin using piperidine in dry dioxane [39] to give the targeted compounds **5a-x** and **6a-h** respectively. The IR spectra of compounds **5a-x** retained the characteristic absorption band of C = O of thiazolidinone at 1719–1685 cm^-1^, while their ^1^H-NMR spectra were characterized by the disappearance of thiazolidinone C^5^-H_2_ singlet at 3.94–4.17 p.p.m. and the appearance of two singlets at 7.51–7.91 and 8.77–8.99 p.p.m. assigned for vinylic -C = CH and pyrazole C^5^-H, respectively. The other protons appeared at their expected chemical shifts. The IR of compounds **6a-h** revealed an additional C = O band at 1687–1663 cm^-1^ assigned for amide I band of 2-oxoindolin together with C = O of thiazolidinone at 1705–1692 cm^-1^. Another absorption band appeared at 3193–3119 cm^-1^ corresponding to the N-H of 2-oxoindolin. In addition, their ^1^H-NMR spectra were characterized by the disappearance of thiazolidinone C^5^-H_2_ singlet at 3.94–4.17 p.p.m. and appearance of deuterium exchangeable singlet at 11.15–11.31 p.p.m. corresponding to N-H of 2-oxoindolin.

During the present investigation, we tried first to obtain our target compounds **5a-x** through condensation of 3-(4-substitutedphenyl)-1-phenyl-1*H*-pyrazole-4-carbaldehydes (**4a-c**) with the active methylene of thiazolidinone ring of compounds (**3a-i**) in acetic acid in the presence of anhydrous sodium acetate according to the previously reported reaction conditions used for the synthesis of analogous compounds [42–44]. Unfortunately, one and the same unexpected product (**7**) was obtained rather than the expected condensed (Figure 4). The IR spectrum of the unexpected product from the condensation of the benzofuran thiazolidinone derivative (**3d**) with the pyrazole aldehyde (**4b**) under the same conditions lacked the C-O-C bands of benzofuran and showed the bands for C = O at 1699 cm^-1^ and C = N at 1619 cm^-1^, while the IR spectrum of the unexpected product from the condensation of the benzimidazole thiazolidinone derivative (**3i**) with the pyrazole aldehyde (**4b**) also lacked the N-H band of benzimidazole at 3333–3241 cm^-1^ and showed the bands for C = O at 1694 cm^-1^ and C = N at 1618 cm^-1^. Comparing the two unexpected products, both were found to have the same melting point, the same R_f_ value in thin layer chromatography and the same elemental analysis. Moreover, the ^1^H-NMR spectra of the unexpected products obtained from the benzimidazole or benzofuran derivatives lacked the aromatic protons of benzimidazole or benzofuran, the singlet corresponding to -N = C-CH_3_ at 2.23–2.60 p.p.m. and the deuterium exchangeable singlet corresponding to N-H of benzimidazole at 11.22–12.56 p.p.m. In addition, the unexpected compounds retained the singlet at 3.94–4.14 p.p.m. attributed to thiazolidinone C^5^-H_2_ and both were also characterized by the appearance of two singlets at 8.30–8.50 and 8.80–8.95 p.p.m. assigned for -CH = N-N = and pyrazole C^5^-H, respectively. Furthermore, the ^1^H-NMR spectra of the two unexpected products obtained from the benzimidazole and benzofuran derivatives were superimposed and stacked indicating they were one and the same compound (Supplementary Figure 1). We assumed that acetic acid resulted in hydrolysis of the hydrazono linkage followed by acid catalyzed condensation of the produced 2-hydrazono-3-cyclohexyl-thiazolidin-4-one with the pyrazole aldehyde (aldehydes are more reactive than ketones toward nucleophiles; Supplementary Figure 2). In addition, during our trials to perform the condensation of thiazolidinone derivatives (**3a-i**) with isatin in acetic acid in the presence of sodium acetate, the benzofuran thiazolidinone (**3d**) was reacted with isatin and the unexpected product (**8**) was obtained in 38% yield (Figure 4). The IR spectrum of the unexpected product (**8**) lacked the C-O-C bands of benzofuran while showing the bands of C = O at 1725 cm^-1^ and C = N at 1608 cm^-1^. The ^1^H-NMR spectrum of compound (**8**) lacked the singlet corresponding to -N = C-CH_3_ at 2.24–2.52 p.p.m. and the singlet corresponding to thiazolidinone C^5^-H_2_ at 3.94–4.14 p.p.m.. The spectrum also showed only eight aromatic protons instead of nine in the expected condensed product. In addition, two deuterium exchangeable singlets appeared at 10.79 and 11.18 p.p.m. instead of one at 11.15–11.24 p.p.m. in the expected condensed product. However, on using two moles of isatin (instead of 1.1 moles) the yield increased to 63%. This was a further confirmation of our conclusion.

### Biological evaluation

#### *In vitro* hemolytic assay

Compounds **5a-x** and **6a-h** were subjected to the *in vitro* hemolytic assay to identify those which cause hemolysis to red blood cells (RBCs). Consequently, these compounds would be toxic *in vivo* so they were excluded from further biological screening. However, compounds **5h, 5s, 5t** and **5u** could not be dissolved in dimethylformamide (DMF) or DMSO under experimental conditions; therefore, these compounds were excluded from biological screening. It was found that compounds **5a-g, 5i-l, 5p-r, 5v-w** and **6b-g** were nontoxic to RBCs, but compounds **5m-o, 5x, 6a** and **6h** were found to cause hemolysis to RBCs, and were excluded from further testing (Supplementary Table 1).

#### *In vitro* anticancer screening

**Figure 5.**
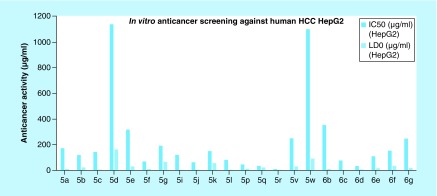
***In vitro* anticancer screening (IC_50_ and LD_0_) against human hepatocellular carcinoma cell line HepG2.** HCC: Hepatocellular carcinoma.

The safe compounds on RBCs (**5a-g, 5i-l, 5p-r, 5v-w, 6b-g**) were screened for their potential anticancer activity against human HCC cell line HepG2. HepG2 cells were exposed to the test compounds, then viability of cells was measured using neutral red uptake assay as described by Borenfreund and Puerner [45]. This assay depends on the fact that neutral red dye can be incorporated into the lysosomes of living cells [46] providing a quantitative assay to the cytotoxic effects. The results were interpreted to calculate both the concentration causing 50% cancer cell death (IC_50_) of each compound and the maximum safe concentration that cause 100% viability (LD_0_; to be used in the *in vitro* anti-HCV testing) using GraphPad InStat 3.0 software (Figure 5 & Supplementary Table 2) [47].

It was reported that IC_50_ values <100 μg/ml reflected a potential anticancer activity, while values between 100 and 1000 μg/ml indicated a moderate anticancer activity [48]. The results revealed that eight compounds showed potential *in vitro* anticancer activity against HepG2 cells with IC_50_ values <100 μg/ml. Compounds **5p, 5q, 5r** and **6d** exhibited the most potent anticancer activity with IC_50_ values <50 μg/ml, while compounds **5f, 5j, 5l** and **6c** showed anticancer activity with IC_50_ values between 50 and 100 μg/ml. The rest of our compounds showed a moderate anticancer activity with IC_50_ values between 100 and 1000 μg/ml, except for compounds **5d** and **5w** which showed a very weak anticancer activity against HepG2 cells.

#### *In vitro* cytotoxicity assay

**Figure 6.**
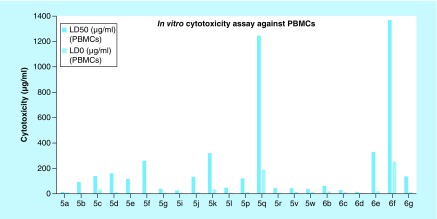
***In vitro* cytotoxicity (LD_50_ and LD_0_) assay against peripheral blood mononuclear cells.** PBMCs: Peripheral blood mononuclear cells.

The safe compounds on RBCs (**5a-g, 5i-l, 5p-r, 5v-w, 6b-g**) were subjected to the *in vitro* cytotoxicity assay on human peripheral blood mononuclear cells (PBMCs). Viability of cells was measured using neutral red uptake assay as described by Borenfreund and Puerner [45] to determine the concentrations of each compound that were not cytotoxic. The results were interpreted to calculate both the lethal concentration that kills 50% of cells (LD_50_) and the maximum safe concentration that causes 100% viability (LD_0_) of each compound using GraphPad InStat 3.0 software (Figure 6 & Supplementary Table 3) [47].

The above results showed that the concentrations that retained 100% viability of the cells (LD_0_) ranged from 4.26 to 33.29 μg/ml. Exceptions were compounds **5q** and **6f** that showed relatively high LD_0_ values.

One of the most important criteria of an anticancer agent is its ability to discriminate between cancer and normal cells. To assess the selectivity of the active newly synthesized compounds, selectivity index was calculated for the compounds which showed potential *in vitro* anticancer effect against HepG2 cells. Selectivity index is a measure of the selectivity of the drug candidate toward cancer cells rather than normal cells (selectivity index = LD_50_ on normal cells/IC_50_ on cancer cells). It was also reported that compounds with selectivity index values larger than three could be considered as highly selective (Table 1) [48].

**Table 1.** **Selectivity index^†^ values for the active *in vitro* anticancer compounds.**

**Compound ID**	**LD_50_ (PBMCs) (μg/ml)**	**IC_50_ (HepG2) (μg/ml)**	**Selectivity index**
**5f**	259.71	69.44	3.74

**5j**	134.00	63.81	2.10

**5l**	46.89	82.08	0.57

**5p**	120.46	46.51	2.58

**5q**	1246.96	35.72	62.90

**5r**	44.00	11.01	3.99

**6c**	28.67	76.82	0.37

**6d**	14.32	34.28	0.42

^†^The selectivity index is the ratio of the LD_50_ values of the treatments on PBMC cells to IC_50_ in the human hepatocellular carcinoma cell lines (HepG2).

PBMC: Peripheral blood mononuclear cell.

Compounds **5f, 5q** and **5r** showed selectivity index values larger than three, and therefore could be considered selective against HepG2 cells. Interestingly, compound **5q** could be considered as a promising anticancer lead compound as it showed a remarkable anticancer effect on HepG2 cancer cells (IC_50_ = 35.72 μg/ml), and a safer effect on PBMCs normal cells (LD_0_ = 190.45 μg/ml). Moreover, the concentration that killed 100% of cancer cells (IC_100_) was calculated for this compound (using GraphPad InStat 3.0 software) [47], and was found to be 94.54 μg/ml. This value was also much lower than the LD_0_ of this compound on PBMCs. The selectivity index of this compound was found to be 62.9. This very high value indicated that compound **5q** was highly selective toward HCC HepG2 cells.

#### *In vitro* anti-HCV screening

HCC HepG2 cell line was found to be the most susceptible cell culture system to HCV infection [49], therefore it could be used to support reliable and efficient progression of HCV. The safe compounds on RBCs (**5a-g, 5i-l, 5p-r, 5v-w, 6b-g**) were screened for their *in vitro* anti-HCV activity using the HCC HepG2 cell line infected with the HCV. Monitoring of the HCV viremia pre- and post-antiviral therapy through the detection of viral RNA using qualitative reverse transcription-PCR (RT-PCR) was adopted in the present investigation. This technique was reported to be the most frequently used, reliable and sensitive technique [50]. Inhibition of viral replication was detected by amplification of viral RNA segments by the RT-PCR technique both in the cultivated infected cells alone (as a positive control) and at the specified dose for each test compound at optimal temperature. The test compound was considered to be active when it can inhibit the viral replication inside the HCV-infected HepG2 cells, as evidenced by the disappearance of the viral RNA-amplified products detected by the RT-PCR (compared with positive control).

The used concentration of each compound was chosen to be lower than the LD_0_ on HepG2 cells. Therefore, 100% viability of HepG2 cells was maintained, to make sure that any inhibitory effect was due to anti-HCV activity rather than cytotoxic activity on HepG2 cells. Moreover, the used concentrations were also chosen to be lower than the LD_0_ on PBMCs. Consequently, these doses could be used for inhibition of HCV in normal cells infected with the virus. The results of the *in vitro* anti-HCV screening were listed in Table 2.

**Table 2.** **Results of the *in vitro* anti-hepatitis C virus screening against HepG2 cells infected with hepatitis C virus.**

**Compound ID**	**(LD_0_) PBMCs (μg/ml**	**(LD_0_) HepG2 (μg/ml)**	**Used dose (μg/ml)**	**Result**
**5a**	11.17	10.15	10.10	Positive

**5b**	5.83	23.02	5.80	Negative

**5c**	32.45	5.88	5.80	Negative

**5d**	11.43	163.55	11.40	Negative

**5e**	4.26	31.12	4.30	Negative

**5f**	5.83	10.39	5.80	Negative

**5g**	8.88	66.72	8.80	Negative

**5i**	7.60	0.05	0.05	Negative

**5j**	9.90	5.08	5.00	Negative

**5k**	33.29	56.74	20.00	Negative

**5l**	8.33	5.11	5.10	Positive

**5p**	12.67	13.32	12.60	Positive

**5q**	190.45	23.12	20.00	Negative

**5r**	7.33	0.18	0.15	Negative

**5v**	13.85	30.89	13.80	Positive

**5w**	16.03	92.40	16.00	Negative

**6b**	18.18	11.07	11.00	Negative

**6c**	11.41	3.40	3.40	Negative

**6d**	5.21	7.31	5.20	Negative

**6e**	19.22	16.75	16.70	Negative

**6f**	253.34	35.33	20.00	Negative

**6g**	14.41	18.62	14.40	Negative

PBMC: Peripheral blood mononuclear cell.

**Figure 7.**
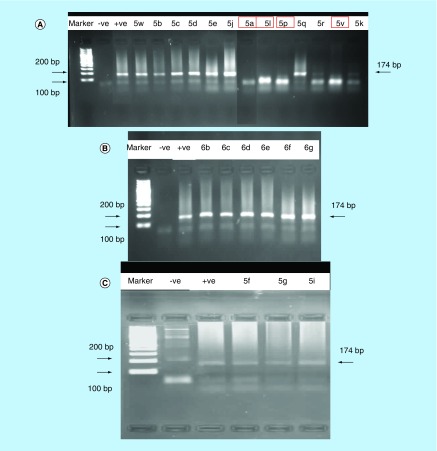
**PCR amplification picture of hepatitis C virus RNA strand in the presence of 13 test compounds.** The first band contains the molecular weight marker, while second and third bands are corresponding to negative and positive controls, respectively. The other bands show the effect of compounds **(A)** **5w, 5b, 5c, 5d, 5e, 5j, 5a, 5l, 5p, 5q, 5r, 5v** and **5k**; **(B)** **6b, 6c, 6d, 6e, 6f** and **6g**; **(C)** **5f, 5g** and **5i**, respectively on hepatitis C virus RNA strand.

The results revealed that four compounds **5a, 5l, 5p** and **5v** exhibited *in vitro* anti-HCV activity, as indicated by the disappearance of the band corresponding to the fragment of 174 base pairs length (Figure 7). These compounds inhibited the virus replication at concentrations ranging from 5.10 to 13.80 μg/ml.

### Structure–activity relationship

Regarding the *in vitro* anticancer activity, it could be noticed that:

- Benzofuran derivatives containing aliphatic cyclohexyl ring as R^1^ appeared to be more active than their corresponding benzimidazoles, while benzimidazole derivatives containing p-Cl-phenyl ring as R^1^ were more active than their corresponding benzofurans. However, no general relation was observed to link different R^1^ substituents to the *in vitro* anticancer activity. This observation might support our rationale that the thiazolidinone nucleus was not a part of the anticancer pharmacophore.
- Some derivatives containing either 1,3-diaryl-1*H*-pyrazole or 2-oxoindolin-3-ylidene pharmacophores exhibited potential *in vitro* anticancer activity. It was observed that the benzofuran derivatives substituted by 2-oxoindolin-3-ylidene were more potent than their corresponding 1,3-diaryl-1*H*-pyrazole analogs. Nevertheless, the benzimidazole derivatives substituted by 2-oxoindolin-3-ylidene were found to be less potent than their 1,3-diaryl-1*H*-pyrazole analogs.
- The substituent R^2^ appeared to influence the anticancer activity of 1,3-diaryl-1*H*-pyrazole derivatives, as the methoxy derivatives were more potent than the chloro derivatives than the unsubstituted ones.
- The combination of methoxy group as R^2^ and p-Cl-phenyl as R^1^ generally increased the anticancer activity.

Regarding the *in vitro* anti-HCV activity, some relations were concluded linking structure to *in vitro* anti-HCV activity.

- The fact that all the anti-HCV compounds had a lipophilic substituent as R^1^, either aliphatic cyclohexyl group (**5l** and **5v**) or aromatic phenyl and p-Cl-phenyl group (**5a** and **5p**), indicated that lipophilicity was an important structure feature regarding HCV inhibition.
- Both benzofuran and benzimidazole derivatives were found to inhibit the virus replication. This confirmed our hypothesis that both scaffolds have aided in the anti-HCV activity.
- An interesting observation was that no compounds containing (2-oxoindolin-3-ylidene) scaffold showed anti-HCV activity, and all the active compounds were 1,3-diaryl-1*H*-pyrazole derivatives. This suggested that 1,3-diaryl-1*H*-pyrazole scaffold played a certain role in HCV inhibition besides its role in HCC suppression.
- The substituent R^2^ appeared to influence the anti-HCV activity of 1,3-diaryl-1*H*-pyrazole derivatives, as unsubstituted and methoxy derivatives were the active compounds.

### Docking studies inside VEGFR-2 active site

**Figure 8.**
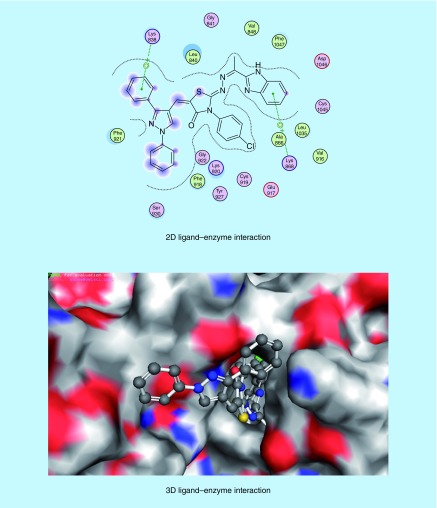
**Mode of binding of compound 5p (IC_50_ = 46.51 μg/ml and docking score = -7.67 kcal/mol, respectively) inside VEGFR-2 active site.**

**Figure 9.**
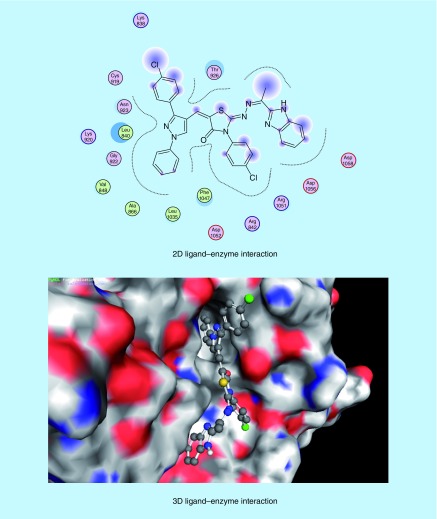
**Mode of binding of compound 5q (IC_50_ = 35.72 μg/ml and docking score = -7.74 kcal/mol) inside VEGFR-2 active site.**

**Figure 10.**
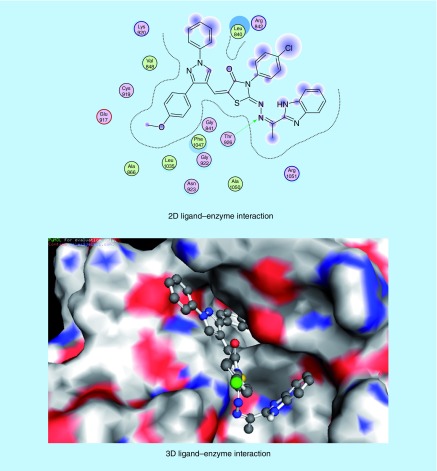
**Mode of binding of compound 5r (IC_50_ = 11.01 μg/ml, docking score = -7.37 kcal/mol) inside VEGFR-2 active site.**

**Figure 11.**
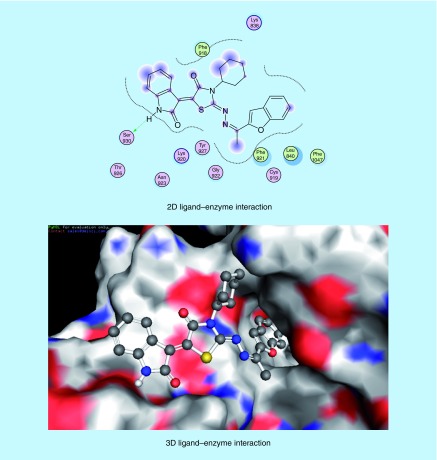
**Mode of binding of compound 6d (IC_50_ = 34.28 μg/ml and docking score = -7.67 kcal/mol) inside VEGFR-2 active site.**

The molecular modeling studies were performed using the Molecular Operating Environment [51] software [52]. The 3D structures and conformations of the enzymes were acquired from the Protein Data Bank (PDB) website [53]. The targeted compounds were docked into the active site of the kinase domain of the VEGFR-2 (PDB ID: 4AGD) to predict their potential kinase inhibition activity. The multikinase inhibitor sunitinib (**VII**) was the co-crystallized ligand, and was also utilized as the reference active drug (Supplementary Table 4) [14,30]. It was found that compounds (**5a-x**) and (**6a-h**) afforded docking scores higher than sunitinib (docking score = -7.14 kcal/mol), in most cases, when docked into the active site of the kinase domain of VEGFR-2. The mode of binding of the most promising *in vitro* anticancer compounds was studied with respect to the type of interactions with the receptor. Docking studies revealed that most of the newly synthesized compounds that exhibited promising *in vitro* anticancer activity mainly interacted with the receptor through the 1,3-diaryl-1*H*-pyrazole (**5p-r**, Figures 8–10) or 2-oxoindolin-3-ylidene (**6d**, Figure 11) scaffolds. 1,3-Diaryl-1*H*-pyrazole moiety appeared to contribute to the hydrophobic interactions with Phe 1047, Leu 840, Ala 866, Val 848 and Leu 1035 residues, as well as arene–cation interactions. In addition, 2-oxoindolin-3-ylidene moiety was responsible mainly for hydrogen bond interaction with Ser 930 residue. It was previously mentioned in our research objectives that 2-oxoindolin-3-ylidene scaffold in the type I inhibitor sunitinib (**VII**) was responsible for hydrogen bond interaction with the hinge region [36], while the pyrazole ring was reported to recognize the ATP-binding site [36] either by forming hydrogen bonds [37], arene–cation interactions or by contribution to the hydrophobic interactions [33,38]. Therefore, docking studies supported our hypothesis that the attachment of well-known kinase inhibitor pharmacophores such as 2-oxoindolin-3-ylidene or 3-aryl-1-phenyl-1*H*-pyrazole might enhance the kinase inhibitory and anticancer activity of our compounds so as to be active against HCC.

## Conclusion

The present study was initiated aiming to design and synthesize novel compounds that exhibit dual activity against HCV and its associated major complication, HCC as an alternative to multidrug therapy. In order to achieve this target, hybrid compounds were designed to incorporate both anti-HCV and anticancer pharmacophores. The anti-HCV pharmacophore was designed to encompass different chemical scaffolds such as benzofuran, benzimidazole and thiazolidinone moieties. In addition, the anticancer pharmacophore was planned to contain moieties like 1,3-diaryl-1*H*-pyrazole or 2-oxoindolin-3-ylidene able to inhibit kinases, specifically tyrosine kinases, to suppress HCC development, angiogenesis and potentiate the anti-HCV activity by inhibition of NS5A activation. Biological screening results revealed that compounds **5f, 5j, 5l, 5p, 5q, 5r, 6c** and **6d** exhibited potential *in vitro* anticancer activity against HCC cell line HepG2, while compounds **5a, 5l, 5p** and **5v** showed *in vitro* anti-HCV activity against HepG2 cells infected with HCV. Consequently, compounds **5l** and **5p** were found to exhibit *in vitro* dual activity against HCV and HCC. The structures of the dual acting compounds (**5l** and **5p**) contained all the planned anti-HCV scaffolds like benzofuran **5l**, benzimidazole **5p** and thiazolidinone (**5l** and **5p**). In addition, all the dual acting compounds contained 1,3-diaryl-1*H*-pyrazole moiety as anticancer pharmacophore rather than 2-oxoindolin-3-ylidene moiety. These suggested that the combination of the planned anti-HCV scaffolds and 1,3-diaryl-1*H*-pyrazole moiety could be considered as an optimal platform for further modification to enhance the dual anti-HCV and anticancer activity. During this work, compound **5q** emerged as a promising anticancer lead compound as it showed a remarkable anticancer effect on HepG2 cancer cells with IC_50_ and IC_100_ values <100 μg/ml and less than its LD_0_ on PBMC normal cells. This compound also showed a very high value of selectivity index indicating high selectivity toward cancerous HepG2 cells. This compound could be considered as a potent, safe and selective anticancer agent against HCC. This compound could be a lead one for further structure modification to achieve more potent anticancer agents. Finally, docking studies suggested that the newly synthesized compounds might suppress HCC through tyrosine kinase inhibition. In addition, inhibiting kinases could inhibit phosphorylation (activation) of HCV NS5A enzyme which might add to the *in vitro* anti-HCV effect of the dual acting compounds.

## Experimental

### Chemistry

Melting points were determined in open glass capillaries on a Stuart SMP10 melting point apparatus (Bibby Scientiﬁc Ltd, Stone, UK) and were uncorrected. IR spectra were recorded, using KBr discs υ(cm^-1^), on a Perkin-Elmer 1430 Infrared spectrophotometer (Central Laboratory, Faculty of Pharmacy, Alexandria University, Egypt). Nuclear magnetic resonance spectra, ^1^H-NMR were scanned on a Jeol NMR 500 MHz spectrophotometer (Faculty of Science, Alexandria University and National Research Center, Dokki, Cairo) and Mercury 300 MHz spectrophotometer (Faculty of Science, Cairo University, Egypt). The data were reported as δ-values (p.p.m.) relative to tetramethylsilane as internal standard. The type of signal was indicated by one of the following letters: s = singlet, d = doublet, t = triplet, q = quartet, m = multiplet, br. = broad, dist. = distorted. Mass spectra [54] were run on a gas chromatograph/mass spectrometer Shimadzu GCMS-Qp2010 plus (70 ev; Faculty of Science, Cairo University, Egypt). The peak intensities, in parentheses, are expressed as percentage abundance. Elemental analyses were performed at the microanalytical unit, Faculty of Pharmacy, Assiut University and at the Regional Center for Mycology and Biotechnology, Al-Azhar University; all the values were within range of ±0.4. Reactions were monitored by thin-layer chromatography on silica gel (60 GF254, Merck, Darmstadt, Germany), using glass plates and the spots were visualized by exposure to iodine vapor or UV-lamp at l 254 nm for few seconds. All reagents and solvents were obtained from commercial sources, and were purified and dried by standard techniques.

Compounds **1a** [55], **1b** [56–61], **2a** [39], **2f** [62], **2g** [62], **2i** [62], **3a** [39] and **4a-c** [63–65] were prepared according to the reported procedures.

#### General procedure for the synthesis of thiosemicarbazones intermediates 2a-i

To a suspension of 2-acetylbenzofuran or 2-acetylbenzimidazole (**1a-b**) (0.8g, 5 mmol) and the appropriately substituted thiosemicarbazide (5 mmol) in 10 ml absolute ethanol, few drops of glacial acetic acid was added. The reaction mixture was refluxed for 3–4 h, then allowed to cool to room temperature. The precipitate formed was filtered, washed with ethanol, air dried and recrystallized from ethanol.

##### 1-[1-(Benzofuran-2-yl)ethylideneamino]-3-(4-chlorophenyl)thiourea 2b

Yellowish white solid, yield 84%, melting point (m.p.) 220–2°C. Fourier-transform infrared spectroscopy (FT-IR) (cm^-1^): 3230 (N-H); 1634 (C = N); 1571, 1304, 1187, 927 (N-C = S amide I, II, III and IV bands); 1262, 1158, 1093 (C-O-C). ^1^H-NMR (DMSO-d_6_, Mercury 300 MHz): δ 2.43 (s, 3H, -N = C-CH_3_); 7.23–7.74 (m, 9H, Ar-C^2,3,5,6^-Hs and benzofuran C^3,4,5,6,7^-Hs); 10.09, 10.93 (2 s, each 1H, 2 -NH of thiosemicarbazone, D_2_O exchangeable). Anal. Calcd. for C_17_H_14_ClN_3_OS: C, 59.38; H, 4.10; N, 12.22. Found: C, 59.51; H, 4.15; N, 12.31.

##### 1-[1-(Benzofuran-2-yl)ethylideneamino]-3-(4-methoxyphenyl)thiourea 2c

Yellowish white solid, yield 87%, m.p. 204–6°C. FT-IR (cm^-1^): 3225 (N-H); 1627 (C = N); 1569, 1302, 1182, 924 (N-C = S amide I, II, III and IV bands); 1259, 1156, 1090 (C-O-C). ^1^H-NMR (DMSO-d_6_, Mercury 300 MHz): δ 2.41 (s, 3H, -N = C-CH_3_); 3.78 (s, 3H, O-CH_3_); 6.94 (d, J = 9 Hz, 2H, Ar-C^2,6^-Hs); 7.24–7.31 (dist. dd, 1H, benzofuran C^5^-H); 7.33–7.39 (dist. dd, 1H, benzofuran C^6^-H); 7.44 (d, J = 9 Hz, 2H, Ar-C^3,5^-Hs); 7.62 (d, J = 8 Hz, 1H, benzofuran C^7^-H); 7.65–7.70 (m, 2H, benzofuran C^3,4^-Hs); 9.91, 10.76 (2 s, each 1H, 2 -NH of thiosemicarbazone, D_2_O exchangeable). Anal. Calcd. for C_18_H_17_N_3_O_2_S: C, 63.70; H, 5.05; N, 12.38. Found: C, 63.83; H, 5.03; N, 12.54.

##### 1-[1-(Benzofuran-2-yl)ethylideneamino]-3-cyclohexylthiourea 2d

White solid, yield 90%, m.p. 182–4°C. FT-IR (cm^-1^): 3218 (N-H); 1629 (C = N); 1568, 1305, 1187, 922 (N-C = S amide I, II, III and IV bands); 1258, 1151, 1091 (C-O-C). ^1^H-NMR (DMSO-d_6_, Mercury 300 MHz): δ 1.12–1.51, 1.54–1.78 and 1.86–1.99 (3 m, 10H, cyclohexyl C^2,3,4,5,6^-H_2_); 2.34 (s, 3H, -N = C-CH_3_); 4.11–4.30 (m, 1H, cyclohexyl C^1^-H); 7.24–7.31 (dist. dd, 1H, benzofuran C^5^-H); 7.32–7.40 (dist. dd, 1H, benzofuran C^6^-H); 7.52 (s, 1H, benzofuran C^3^-H); 7.58–7.63 (dist. d, 1H, benzofuran C^7^-H); 7.66–7.71 (dist. d, 1H, benzofuran C^4^-H); 7.99 (d, J = 8.5 Hz, 1H, -NH-cyclohexyl, D_2_O exchangeable); 10.43 (s, 1H, -NH of thiosemicarbazide, D_2_O exchangeable). Anal. Calcd. for C_17_H_21_N_3_OS.1/2 H_2_O: N, 12.95; S, 9.88. Found: N, 13.20; S, 9.47.

##### 1-[1-(1H-Benzimidazole-2-yl)ethylideneamino]-3-phenylthiourea 2e

White solid, yield 92%, m.p. 176–8°C. FT-IR (cm^-1^): 3340 (N-H); 1625 (C = N); 1569, 1301, 1184, 923 (N-C = S amide I, II, III and IV bands). ^1^H-NMR (DMSO-d_6_, Mercury 300 MHz): δ 2.53 (s, 3H, -N = C-CH_3_); 7.16–7.59 (m, 8H, benzimidazole C^4,5,6^-Hs and Ar-C^2,3,4,5,6^-Hs); 7.70 (d, J = 7.8 Hz, benzimidazole C^7^-H); 10.43, 11.02 (2 s, each 1H, 2 -NH of thiosemicarbazone, D_2_O exchangeable); 12.84 (s, 1H, -NH of benzimidazole, D_2_O exchangeable). Anal. Calcd. for C_16_H_15_N_5_S: C, 62.11; H, 4.89; N, 22.64. Found: C, 62.17; H, 4.93; N, 22.81.

##### 1-[1-(1H-Benzimidazole-2-yl)ethylideneamino]-3-(4-methylphenyl)thiourea 2h

Yellowish white solid, yield 83%, m.p. 184–6°C. FT-IR (cm^-1^): 3325 (N-H); 1618 (C = N); 1563, 1298, 1190, 926 (N-C = S amide I, II, III and IV bands). Anal. Calcd. for C_17_H_17_N_5_S: C, 63.13; H, 5.30; N, 21.65. Found: C, 63.22; H, 5.28; N, 21.78.

#### General procedure for the synthesis of 4-thiazolidinone intermediates 3a-i

A mixture of 1-[1-(benzofuran-2-yl)ethylideneamino]-3-substituted thioureas or 1-[1-(1*H*-benzimidazol-2-yl)ethylideneamino]-3-substituted thioureas **(2a-i)** (5 mmol), monochloroacetic acid (0.65 g, 7.5 mmol) and anhydrous sodium acetate (0.62 g, 7.5 mmol) in glacial acetic acid (10 ml) was heated under reflux for 5–6 h, left to attain room temperature, then poured onto crushed ice. The precipitate formed was filtered, washed with water and crystallized from ethanol.

##### 2-[1-(Benzofuran-2-yl)ethylidenehydrazono]-3-(4-chlorophenyl)thiazolidin-4-one 3b

Yellow solid, yield 72%, m.p. 254–6°C. FT-IR (cm^-1^): 1726 (C = O); 1606 (C = N); 1259, 1171, 1087 (C-O-C). ^1^H-NMR (DMSO-d_6_, Mercury 300 MHz): δ 2.18 (s, 3H, -N = C-CH_3_); 4.11 (s, 2H, thiazolidinone C^5^-H_2_); 7.25–7.32 (dist. dd, 1H, benzofuran C^5^-H); 7.36–7.42 (dist. dd, 1H, benzofuran C^6^-H); 7.44 (s, 1H, benzofuran C^3^-H); 7.48 (d, J = 8.6 Hz, Ar-C^2,6^-Hs); 7.61 (d, J = 8.6 Hz, Ar-C^3,5^-Hs); 7.65 (d, J = 7.7 Hz, 1H, benzofuran C^7^-H); 7.71 (d, J = 7.7 Hz, 1H, benzofuran C^4^-H). Anal. Calcd. for C_19_H_14_ClN_3_O_2_S: C, 59.45; H, 3.68; N, 10.95. Found: C, 59.59; H, 3.74; N, 11.12.

##### 2-[1-(Benzofuran-2-yl)ethylidenehydrazono]-3-(4-methoxyphenyl)thiazolidin-4-one 3c

Yellow solid, yield 73%, m.p. 239–41°C. FT-IR (cm^-1^): 1722 (C = O); 1610 (C = N); 1257, 1170, 1082 (C-O-C). ^1^H-NMR (DMSO-d_6_, Mercury 300 MHz): δ 2.18 (s, 3H, -N = C-CH_3_); 3.82 (s, 3H, O-CH_3_); 4.09 (s, 2H, thiazolidinone C^5^-H_2_); 7.06 (d, J = 9 Hz, 2H, Ar-C^3,5^-Hs); 7.25–7.41 (m, 4H, benzofuran C^5,6^-Hs and Ar-C^2,6^-Hs); 7.42 (s, 1H, benzofuran C^3^-H); 7.65 (d, J = 8 Hz, 1H, benzofuran C^7^-H); 7.71 (d, J = 8 Hz, 1H, benzofuran C^4^-H). Anal. Calcd. for C_20_H_17_N_3_O_3_S: C, 63.31; H, 4.52; N, 11.07. Found: C, 63.44; H, 4.59; N, 11.32.

##### 2-[1-(Benzofuran-2-yl)ethylidenehydrazono]-3-cyclohexylthiazolidin-4-one 3d

Yellow solid, yield 97%, m.p. 172–4°C. FT-IR (cm^-1^): 1724 (C = O); 1609 (C = N); 1256, 1173, 1083 (C-O-C). ^1^H-NMR (DMSO-d_6_, Mercury 300 MHz): δ 1.01–1.45, 1.57–1.72, 1.77–1.90 and 2.28–2.42 (4m, 10H, cyclohexyl C^2,3,4,5,6^-H_2_); 2.43 (s, 3H, -N = C-CH_3_); 3.94 (s, 2H, thiazolidinone C^5^-H_2_); 4.27–4.40 (m, 1H, cyclohexyl C^1^-H); 7.25–7.32 (dist. dd, 1H, benzofuran C^5^-H); 7.35–7.43 (dist. dd, 1H, benzofuran C^6^-H); 7.47 (s, 1H, benzofuran C^3^-H); 7.66 (d, J = 8 Hz, 1H, benzofuran C^7^-H); 7.72 (d, J = 8 Hz, 1H, benzofuran C^4^-H). Anal. Calcd. for C_19_H_21_N_3_O_2_S: C, 64.20; H, 5.95; N, 11.82; S, 9.02. Found: C, 64.19; H, 5.83; N, 11.75; S, 8.71.

##### 2-[1-(1H-Benzimidazole-2-yl)ethylidenehydrazono]-3-phenylthiazolidin-4-one 3e

Yellow solid, yield 81%, m.p. 258–60°C. FT-IR (cm^-1^): 3066 (N-H); 1728 (C = O); 1598 (C = N). ^1^H-NMR (DMSO-d_6_, Mercury 300 MHz): δ 2.26 (s, 3H, -N = C-CH_3_); 4.17 (s, 2H, thiazolidinone C^5^-H_2_); 7.18–7.29 (m, 2H, benzimidazole C^5,6^-Hs); 7.38–7.55 (m, 5H, Ar-C^2,3,4,5,6^-Hs); 7.59–7.64 (m, 2H, benzimidazole C^4,7^-Hs); 12.50 (s, 1H, -NH of benzimidazole, D_2_O exchangeable). Anal. Calcd. for C_18_H_15_N_5_OS: C, 61.87; H, 4.33; N, 20.04. Found: C, 61.49; H, 4.39; N, 20.20.

##### 2-[1-(1H-Benzimidazole-2-yl)ethylidenehydrazono]-3-(4-chlorophenyl)thiazolidin-4-one 3f

Yellow solid, yield 65%, m.p. 249–51°C. FT-IR (cm^-1^): 3050 (N-H); 1725 (C = O); 1601 (C = N). ^1^H-NMR (DMSO-d_6_, Mercury 300 MHz): δ 2.28 (s, 3H, -N = C-CH_3_); 4.16 (s, 2H, thiazolidinone C^5^-H_2_); 7.21–7.28 (m, 2H, benzimidazole C^5,6^-Hs); 7.50 (d, J = 8.7 Hz, 2H, Ar-C^2,6^-Hs); 7.58–7.65 (m, 4H, Ar-C^3,5^-Hs and benzimidazole C^4,7^-Hs); 12.50 (s, 1H, -NH of benzimidazole, D_2_O exchangeable). Anal. Calcd. for C_18_H_14_ClN_5_OS.1/2H_2_O: C, 55.03; H, 3.85; N, 17.83; S, 8.16. Found: C, 55.12; H, 4.03; N, 17.74; S, 8.10.

##### 2-[1-(1H-Benzimidazole-2-yl)ethylidenehydrazono]-3-(4-methoxyphenyl)thiazolidin-4-one 3g

Yellow solid, yield 84%, m.p. 253–5°C. FT-IR (cm^-1^): 3044 (N-H); 1729 (C = O); 1603 (C = N). ^1^H-NMR (DMSO-d_6_, Mercury 300 MHz): δ 2.27 (s, 3H, -N = C-CH_3_); 3.81 (s, 3H, O-CH_3_); 4.14 (s, 2H, thiazolidinone C^5^-H_2_); 7.06 (d, J = 8.5 Hz, 2H, Ar-C^3,5^-Hs); 7.16–7.23 (dist. dd, 1H, benzimidazole C^5^-H); 7.24–7.31 (dist. dd, 1H, benzimidazole C^6^-H); 7.35 (d, J = 8.5 Hz, 2H, Ar-C^2,6^-Hs); 7.56 (d, J = 8 Hz, 1H, benzimidazole C^4^-H); 7.68 (d, J = 8 Hz, 1H, benzimidazole C^7^-H); 12.50 (s, 1H, -NH of benzimidazole, D_2_O exchangeable). Anal. Calcd. for C_19_H_17_N_5_O_2_S: C, 60.14; H, 4.52; N, 18.46; S, 8.45. Found: C, 60.05; H, 4.46; N, 18.31; S, 8.44.

##### 2-[1-(1H-Benzimidazole-2-yl)ethylidenehydrazono]-3-(4-methylphenyl)thiazolidin-4-one 3h

Yellow solid, yield 85%, m.p. 251–3°C. FT-IR (cm^-1^): 3052 (N-H); 1727 (C = O); 1609 (C = N). ^1^H-NMR (CDCl_3_, Jeol 500 MHz): δ 1.59 (s, 3H, p-CH_3_ phenyl); 2.43 (s, 3H, -N = C-CH_3_); 4.01 (s, 2H, thiazolidinone C^5^-H_2_); 7.26–7.50 (m, 8H, benzimidazole C^4,5,6,7^-Hs and Ar-C^2,3,5,6^-Hs); 10.12 (s, 1H, -NH of benzimidazole, D_2_O exchangeable).   Electron-impact mass spectrum (EIMS) m/z (% abundance), 365 (4.10) M^+^ + 2; 364 (13.56) M^+^ +1; 363 (51.62) M^+^; 118 (100). Anal. Calcd. for C_19_H_17_N_5_OS: C, 62.79; H, 4.71; N, 19.27. Found: C, 62.88; H, 4.69; N, 19.41.

##### 2-[1-(1H-Benzimidazole-2-yl)ethylidenehydrazono]-3-cyclohexylthiazolidin-4-one 3i

Yellow solid, yield 83%, m.p. 229–31°C. FT-IR (cm^-1^): 3035 (N-H); 1724 (C = O); 1599 (C = N). ^1^H-NMR (DMSO-d_6_, Mercury 300 MHz): δ 1.12–1.42, 1.59–1.72, 1.78–1.90 and 2.28–2.45 (4m, 10H, cyclohexyl C^2,3,4,5,6^-H_2_); 2.53 (s, 3H, -N = C-CH_3_); 4.00 (s, 2H, thiazolidinone C^5^-H_2_); 4.27–4.42 (m, 1H, cyclohexyl C^1^-H); 7.16–7.24 (dist. dd, 1H, benzimidazole C^5^-H); 7.24–7.31 (dist. dd, 1H, benzimidazole C^6^-H); 7.56 (d, J = 8 Hz, 1H, benzimidazole C^7^-H); 7.69 (d, J = 8 Hz, 1H, benzimidazole C^4^-H); 12.43 (s, 1H, -NH of benzimidazole, D_2_O exchangeable). Anal. Calcd. for C_18_H_21_N_5_OS: C, 60.82; H, 5.95; N, 19.70; S, 9.02. Found: C, 60.95; H, 5.75; N, 19.54; S, 8.81.

#### General procedure for the synthesis of compounds 5a-x

To a mixture of 2-[1-(benzofuran-2-yl)ethylidenehydrazono]-3-substituted thiazolidin-4-ones or 2-[1-(1*H*-benzimidazol-2-yl)ethylidenehydrazono]-3-substituted thiazolidin-4-ones **(3a-i)** (1 mmol) and 3-(4-substituted phenyl)-1-phenyl-1*H*-pyrazole-4-carbaldehydes (**4a-c**) (1.1 mmol) in dry dioxane (5 ml), two drops of piperidine were added. The reaction mixture was refluxed for 8–15 h, concentrated, then allowed to cool to room temperature. The precipitate formed after addition of ethanol (10 ml) was filtered, washed with ethanol and recrystallized from dioxane/ethanol.

##### 2-[1-(Benzofuran-2-yl)ethylidenehydrazono]-5-[(1,3-Diphenyl-1H-pyrazol-4-yl)methylene]-3-phenylthiazolidin-4-one 5a

Yellow solid, yield 80%, m.p. 204–6°C. FT-IR (cm^-1^): 1700 (C = O); 1599 (C = N); 1221, 1166, 1070 (C-O-C). ^1^H-NMR (DMSO-d_6_, Mercury 300 MHz): δ 2.25 (s, 3H, -N = C-CH_3_); 7.24–7.75 (m, 19H, benzofuran C^3,4,5,6,7^-Hs, C^2,3,4,5,6^-Hs of thiazolidinone N^3^-phenyl, C^3,4,5^-Hs of pyrazole C^3^-phenyl, C^2,3,4,5,6^-Hs of pyrazole N^1^-phenyl and -C = CH vinylic); 8.05 (d, J = 8.2 Hz, 2H, C^2,6^-Hs of pyrazole C^3^-phenyl); 8.95 (s, 1H, pyrazole C^5^-H). Anal. Calcd. for C_35_H_25_N_5_O_2_S: C, 72.52; H, 4.35; N, 12.08. Found: C, 72.64; H, 4.41; N, 12.21.

##### 2-[1-(Benzofuran-2-yl)ethylidenehydrazono]-5-{[3-(4-chlorophenyl)-1-phenyl-1H-pyrazol-4-yl]methylene}-3-phenylthiazolidin-4-one 5b

Yellow solid, yield 77%, m.p. 237–9°C. FT-IR (cm^-1^): 1703 (C = O); 1598 (C = N); 1222, 1166, 1075 (C-O-C). ^1^H-NMR (DMSO-d_6_, Mercury 300 MHz): δ 2.24 (s, 3H, -N = C-CH_3_); 7.26–7.75 (m, 18H, benzofuran C^3,4,5,6,7^-Hs, C^2,3,4,5,6^-Hs of thiazolidinone N^3^-phenyl, C^2,3,4,5,6^-Hs of pyrazole N^1^-phenyl, C^3,5^-Hs of p-Cl-phenyl ring and -C = CH vinylic); 8.08 (d, J = 8 Hz, 2H, C^2,6^-Hsof p-Cl-phenyl ring); 8.96 (s, 1H, pyrazole C^5^-H). Anal. Calcd. for C_35_H_24_ClN_5_O_2_S: C, 68.45; H, 3.94; N, 11.40. Found: C, 68.53; H, 3.90; N, 11.56.

##### 2-[1-(Benzofuran-2-yl)ethylidenehydrazono]-5-{[3-(4-methoxyphenyl)-1-phenyl-1H-pyrazol-4-yl]methylene}-3-phenylthiazolidin-4-one 5c

Yellow solid, yield 90%, m.p. 187–9°C. FT-IR (cm^-1^): 1704 (C = O); 1601 (C = N); 1225, 1170, 1079 (C-O-C). ^1^H-NMR (DMSO-d_6_, Mercury 300 MHz): δ 2.24 (s, 3H, -N = C-CH_3_); 3.83 (s, 3H, O-CH_3_); 7.12 (d, J = 8.5 Hz, 2H, C^3,5^-Hs of p-OCH_3_-phenyl ring); 7.26–7.36 (dist. dd, 1H, benzofuran C^5^-H); 7.37–7.71 (m, 14H, benzofuran C^3,6,7^-Hs, C^2,3,4,5,6^-Hs of thiazolidinone N^3^-phenyl, C^2,3,4,5,6^-Hs of pyrazole N^1^-phenyl and -C = CH vinylic); 7.73 (d, J = 7.5 Hz, 1H, benzofuran C^4^-H); 8.06 (d, J = 8.5 Hz, 2H, C^2,6^-Hs of p-OCH_3_-phenyl ring); 8.90 (s, 1H, pyrazole C^5^-H). Anal. Calcd. for C_36_H_27_N_5_O_3_S: C, 70.92; H, 4.46; N, 11.49. Found: C, 70.95; H, 4.52; N, 11.58.

##### 2-[1-(Benzofuran-2-yl)ethylidenehydrazono]-5-[(1,3-Diphenyl-1H-pyrazol-4-yl)methylene]-3-(4-chlorophenyl)thiazolidin-4-one 5d

Yellow solid, yield 89%, m.p. 242–4°C. FT-IR (cm^-1^): 1714 (C = O); 1609 (C = N); 1230, 1178, 1081 (C-O-C). ^1^H-NMR (DMSO-d_6_, Mercury 300 MHz): δ 2.27 (s, 3H, -N = C-CH_3_); 7.25–7.84 (m, 18H, benzofuran C^3,4,5,6,7^-Hs, C^2,3,4,5,6^-Hs of pyrazole N^1^-phenyl, C^2,3,5,6^-Hs of p-Cl-phenyl ring, C^3,4,5^-Hs of pyrazole C^3^-phenyl and -C = CH vinylic); 8.09 (d, J = 8 Hz, 2H, C^2,6^-Hs of pyrazole C^3^-phenyl); 8.96 (s, 1H, pyrazole C^5^-H). Anal. Calcd. for C_35_H_24_ClN_5_O_2_S: C, 68.45; H, 3.94; N, 11.40. Found: C, 68.55; H, 3.98; N, 11.57.

##### 2-[1-(Benzofuran-2-yl)ethylidenehydrazono]-5-{[3-(4-chlorophenyl)-1-phenyl-1H-pyrazol-4-yl]methylene}-3-(4-chlorophenyl)thiazolidin-4-one 5e

Yellow solid, yield 79%, m.p. 247–9°C. FT-IR (cm^-1^): 1712 (C = O); 1611 (C = N); 1230, 1180, 1075 (C-O-C). ^1^H-NMR (DMSO-d_6_, Mercury 300 MHz): δ 2.26 (s, 3H, -N = C-CH_3_); 7.26–7.37 (dist. dd, 1H, benzofuran C^5^-H); 7.37–7.52 (m, 2H, benzofuran C^6^-H and C^4^-H of pyrazole N^1^-phenyl); 7.57–7.76 (m, 14H, benzofuran C^3,4,7^-Hs, C^2,3,5,6^-Hs of pyrazole N^1^-phenyl, C^2,3,5,6^-Hs of p-Cl-phenyl ring of thiazolidinone, C^3,5^-Hs of p-Cl-phenyl ring of pyrazole and -C = CH vinylic); 8.08 (d, J = 8 Hz, 2H, C^2,6^-Hs of p-Cl-phenyl ring of pyrazole); 8.96 (s, 1H, pyrazole C^5^-H). Anal. Calcd. for C_35_H_23_Cl_2_N_5_O_2_S: C, 64.82; H, 3.57; N, 10.80. Found: C, 64.97; H, 3.59; N, 10.92.

##### 2-[1-(Benzofuran-2-yl)ethylidenehydrazono]-5-{[3-(4-methoxyphenyl)-1-phenyl-1H-pyrazol-4-yl]methylene}-3-(4-chlorophenyl)thiazolidin-4-one 5f

Yellow solid, yield 89%, m.p. 226–8°C. FT-IR (cm^-1^): 1720 (C = O); 1618 (C = N); 1227, 1183, 1078 (C-O-C). ^1^H-NMR (DMSO-d_6_, Mercury 300 MHz): δ 2.26 (s, 3H, -N = C-CH_3_); 3.83 (s, 3H, O-CH_3_); 7.13 (d, J = 8.5 Hz, 2H, C^3,5^-Hs of p-OCH_3_-phenyl ring); 7.31 (dist. dd, 1H, C^4^-H of pyrazole N^1^-phenyl); 7.39–7.47 (m, 2H, benzofuran C^5,6^-Hs); 7.55–7.66 (m, 9H, C^2,3,5,6^-Hs of pyrazole N^1^-phenyl, C^2,6^-Hs of p-Cl-phenyl ring, benzofuran C^3,7^-Hs and -C = CH vinylic); 7.69 (d, J = 8.6 Hz, 2H, C^3,5^-Hs of p-Cl-phenyl ring); 7.71–7.78 (m, 1H, benzofuran C^4^-H); 8.07 (d, J = 8.5 Hz, 2H, C^2,6^-Hs of p-OCH_3_-phenyl ring); 8.90 (s, 1H, pyrazole C^5^-H). Anal. Calcd. for C_36_H_26_ClN_5_O_3_S: C, 67.13; H, 4.07; N, 10.87. Found: C, 67.20; H, 4.11; N, 10.99.

##### 2-[1-(Benzofuran-2-yl)ethylidenehydrazono]-5-[(1,3-Diphenyl-1H-pyrazol-4-yl)methylene]-3-(4-methoxyphenyl)thiazolidin-4-one 5g

Yellow solid, yield 81%, m.p. 207–9°C. FT-IR (cm^-1^): 1719 (C = O); 1597 (C = N); 1234, 1179, 1079 (C-O-C). ^1^H-NMR (DMSO-d_6_, Mercury 300 MHz): δ 2.26 (s, 3H, -N = C-CH_3_); 3.83 (s, 3H, O-CH_3_); 7.09 (d, J = 9 Hz, 2H, C^3,5^-Hs of p-OCH_3_-phenyl ring); 7.27–7.35 (dist. dd, 1H, benzofuran C^5^-H); 7.38–7.47 (m, 4H, benzofuran C^6^-H, C^2,6^-Hs of p-OCH_3_-phenyl ring and C^4^-H of pyrazole C^3^-phenyl); 7.51–7.71 (m, 10H, benzofuran C^3,7^-Hs, C^2,3,4,5,6^-Hs of pyrazole N^1^-phenyl, C^3,5^-Hs of pyrazole C^3^-phenyl and -C = CHvinylic); 7.73 (d, J = 7.5 Hz, 1H, benzofuran C^4^-H); 8.08 (d, J = 8 Hz, 2H, C^2,6^-Hs of pyrazole C^3^-phenyl); 8.93 (s, 1H, pyrazole C^5^-H). Anal. Calcd. for C_36_H_27_N_5_O_3_S.1/2H_2_O: C, 69.89; H, 4.56; N, 11.32; S, 5.18. Found: C, 69.60; H, 4.42; N, 11.14; S, 4.82.

##### 2-[1-(Benzofuran-2-yl)ethylidenehydrazono]-5-{[3-(4-chlorophenyl)-1-phenyl-1H-pyrazol-4-yl]methylene}-3-(4-methoxyphenyl)thiazolidin-4-one 5h

Yellow solid, yield 69%, m.p. 280–2°C. FT-IR (cm^-1^): 1706 (C = O); 1592 (C = N); 1237, 1185, 1073 (C-O-C). ^1^H-NMR (DMSO-d_6_, Mercury 300 MHz): δ 2.26 (s, 3H, -N = C-CH_3_); 3.84 (s, 3H, O-CH_3_); 7.09 (d, J = 9 Hz, 2H, C^3,5^-Hs of p-OCH_3_-phenyl ring); 7.26–7.36 (dist. dd, 1H, benzofuran C^5^-H); 7.39–7.47 (m, 4H, benzofuran C^6^-H, C^2,6^-Hs of p-OCH_3_-phenyl ring and C^4^-H of pyrazole N^1^-phenyl); 7.55–7.76 (m, 10H, benzofuran C^3,4,7^-Hs, C^2,3,5,6^-Hs of pyrazole N^1^-phenyl, C^3,5^-Hs of p-Cl-phenyl ring and -C = CH vinylic); 8.07 (d, J = 8 Hz, 2H, C^2,6^-Hs of p-Cl-phenyl ring); 8.95 (s, 1H, pyrazole C^5^-H). EIMS m/z (% abundance), 644 (76.12) M^+^ + 1; 643 (52.24) M^+^; 189 (100). Anal. Calcd. for C_36_H_26_ClN_5_O_3_S: N, 10.87; S, 4.98. Found: N, 10.57; S, 5.03.

##### 2-[1-(Benzofuran-2-yl)ethylidenehydrazono]-5-{[3-(4-methoxyphenyl)-1-phenyl-1H-pyrazol-4-yl]methylene}-3-(4-methoxyphenyl)thiazolidin-4-one 5i

Yellow solid, yield 89%, m.p. 282–4°C. FT-IR (cm^-1^): 1702 (C = O); 1596 (C = N); 1241, 1179, 1071 (C-O-C). ^1^H-NMR (DMSO-d_6_, Mercury 300 MHz): δ 2.26 (s, 3H, -N = C-CH_3_); 3.90 (s, 6H, 2 O-CH_3_); 7.06–7.18 (m, 4H, C^3,5^-Hs of p-OCH_3_-phenyl ring of thiazolidinone and C^3,5^-Hs of p-OCH_3_-phenyl ring of pyrazole); 7.28–7.34 (dist. dd, 1H, benzofuran C^5^-H); 7.38–7.51 (m, 4H, benzofuran C^6^-H, C^2,6^-Hs of p-OCH_3_-phenyl ring of thiazolidinone & C^4^-H of pyrazole N^1^-phenyl); 7.52–7.66 (m, 6H, C^2,3,5,6^-Hs of pyrazole N^1^-phenyl, benzofuran C^3^-H and -C = CH vinylic); 7.67–7.76 (m, 2H, benzofuran C^4,7^-Hs); 8.07 (d, J = 7.5 Hz, 2H, C^2,6^-Hsof p-OCH_3_-phenyl ring of pyrazole); 8.90 (s, 1H, pyrazole C^5^-H). Anal. Calcd. for C_37_H_29_N_5_O_4_S.1/2H_2_O: C, 68.50; H, 4.66; N, 10.80; S, 4.94. Found: C, 68.82; H, 4.46; N, 10.79; S, 4.88.

##### 2-[1-(Benzofuran-2-yl)ethylidenehydrazono]-5-[(1,3-Diphenyl-1H-pyrazol-4-yl)methylene]-3-cyclohexylthiazolidin-4-one 5j

Yellow solid, yield 56%, m.p. 187–9°C. FT-IR (cm^-1^): 1704 (C = O); 1606 (C = N); 1247, 1177, 1080 (C-O-C). ^1^H-NMR (DMSO-d_6_, Mercury 300 MHz): δ 1.08–1.49, 1.62–1.94 and 2.31–2.45 (3m, 10H, cyclohexyl C^2,3,4,5,6^-H_2_); 2.51 (s, 3H, -N = C-CH_3_); 4.43–4.57 (m, 1H, cyclohexyl C^1^-H); 7.26–7.36 (dist. dd, 1H, benzofuran C^5^-H); 7.39–7.46 (m, 2H, benzofuran C^6^-H and C^4^-H of pyrazole N^1^-phenyl); 7.49–7.72 (m, 10H, benzofuran C^3,7^-Hs, C^2,3,5,6^-Hs of pyrazole N^1^-phenyl, C^3,4,5^-Hs of pyrazole C^3^-phenyl and -C = CH vinylic); 7.74 (d, J = 7.5 Hz, 1H, benzofuran C^4^-H); 8.05 (d, J = 8 Hz, 2H, C^2,6^-Hs of pyrazole C^3^-phenyl); 8.85 (s, 1H, pyrazole C^5^-H). Anal. Calcd. for C_35_H_31_N_5_O_2_S: C, 71.77; H, 5.33; N, 11.96. Found: C, 71.81; H, 5.35; N, 12.14.

##### 2-[1-(Benzofuran-2-yl)ethylidenehydrazono]-5-{[3-(4-chlorophenyl)-1-phenyl-1H-pyrazol-4-yl]methylene}-3-cyclohexylthiazolidin-4-one 5k

Yellow solid, yield 57%, m.p. 210–2°C. FT-IR (cm^-1^): 1711 (C = O); 1608 (C = N); 1247, 1185, 1079 (C-O-C). ^1^H-NMR (DMSO-d_6_, Mercury 300 MHz): δ 1.08–1.47, 1.61–1.99 and 2.33–2.46 (3m, 10H, cyclohexyl C^2,3,4,5,6^-H_2_); 2.52 (s, 3H, -N = C-CH_3_); 4.40–4.61 (m, 1H, cyclohexyl C^1^-H); 7.29–7.35 (dist. dd, 1H, benzofuran C^5^-H); 7.39–7.47 (m, 2H, benzofuran C^6^-H and C^4^-H of pyrazole N^1^-phenyl); 7.52 (s, 1H, benzofuran C^3^-H); 7.57–7.73 (m, 8H, benzofuran C^7^-H, C^2,3,5,6^-Hs of pyrazole N^1^-phenyl, C^3,5^-Hs of p-Cl-phenyl ring and -C = CH vinylic); 7.74 (d, J = 7.5 Hz, 1H, benzofuran C^4^-H); 8.05 (d, J = 8 Hz, 2H, C^2,6^-Hs of p-Cl-phenyl ring); 8.88 (s, 1H, pyrazole C^5^-H). Anal. Calcd. for C_35_H_30_ClN_5_O_2_S: C, 67.78; H, 4.88; N, 11.29. Found: C, 67.68; H, 4.92; N, 11.41.

##### 2-[1-(Benzofuran-2-yl)ethylidenehydrazono]-5-{[3-(4-methoxyphenyl)-1-phenyl-1H-pyrazol-4-yl]methylene}-3-cyclohexylthiazolidin-4-one 5l

Yellow solid, yield 93%, m.p. 232–4°C. FT-IR (cm^-1^): 1712 (C = O); 1601 (C = N); 1245, 1184, 1073 (C-O-C). ^1^H-NMR (DMSO-d_6_, Mercury 300 MHz): δ 1.15–1.49, 1.69–1.99 and 2.37–2.46 (3m, 10H, cyclohexyl C^2,3,4,5,6^-H_2_); 2.54 (s, 3H, -N = C-CH_3_); 3.84 (s, 3H, O-CH_3_); 4.46–4.61 (m, 1H, cyclohexyl C^1^-H); 7.12 (d, J = 8.5 Hz, 2H, C^3,5^-Hs of p-OCH_3_-phenyl ring); 7.31–7.37 (dist. dd, 1H, benzofuran C^5^-H); 7.38–7.43 (dist. dd, 1H, benzofuran C^6^-H); 7.44–7.50 (dist. dd, 1H, C^4^-H of pyrazole N^1^-phenyl); 7.52 (s, 1H, benzofuran C^3^-H); 7.53–7.60 (m, 4H, C^2,3,5,6^-Hs of pyrazole N^1^-phenyl); 7.66 (d, J = 8 Hz, 1H, benzofuran C^7^-H); 7.74 (d, J = 8 Hz, 1H, benzofuran C^4^-H); 7.91 (s, 1H, -C = CH vinylic); 8.02 (d, J = 8.3 Hz, 2H, C^2,6^-Hs of p-OCH_3_-phenyl ring); 8.76 (s, 1H, pyrazole C^5^-H). Anal. Calcd. for C_36_H_33_N_5_O_3_S: C, 70.22; H, 5.40; N, 11.37. Found: C, 70.35; H, 5.46; N, 11.49.

##### 2-[1-(1H-Benzimidazole-2-yl)ethylidenehydrazono]-5-[(1,3-Diphenyl-1H-pyrazol-4-yl)methylene]-3-phenylthiazolidin-4-one 5m

Yellow solid, yield 55%, m.p. >300°C. FT-IR (cm^-1^): 3333 (N-H); 1718 (C = O); 1623 (C = N). ^1^H-NMR (DMSO-d_6_, Jeol 500 MHz): δ 2.49 (s, 3H, -N = C-CH_3_); 7.08–7.16 (m, 1H, C^4^-H of thiazolidinone N^3^-phenyl); 7.18–7.25 (dist. dd, 1H, benzimidazole-C^5^-H); 7.26–7.33 (dist. dd, 1H, benzimidazole-C^6^-H); 7.40–7.75 (m, 15H, benzimidazole C^4,7^-Hs, C^2,3,5,6^-Hs of thiazolidinone N^3^-phenyl, C^3,4,5^-Hs of pyrazole C^3^-phenyl, C^2,3,4,5,6^-Hs of pyrazole N^1^-phenyl and -C = CH vinylic); 8.08 (d, J = 8 Hz, 2H, C^2,6^-Hs of pyrazole C^3^-phenyl); 8.97 (s, 1H, pyrazole C^5^-H); 11.21 (s, 1H, -NH of benzimidazole, D_2_O exchangeable). Anal. Calcd. for C_34_H_25_N_7_OS: C, 70.45; H, 4.35; N, 16.91. Found: C, 70.49; H, 4.42; N, 17.04.

##### 2-[1-(1H-Benzimidazole-2-yl)ethylidenehydrazono]-5-{[3-(4-chlorophenyl)-1-phenyl-1H-pyrazol-4-yl]methylene}-3-phenylthiazolidin-4-one 5n

Yellow solid, yield 57%, m.p. >300°C. FT-IR (cm^-1^): 3325 (N-H); 1715 (C = O); 1620 (C = N). ^1^H-NMR (DMSO-d_6_, Mercury 300 MHz): δ 2.57 (s, 3H, -N = C-CH_3_); 7.10–7.17 (m, 1H, C^4^-H of thiazolidinone N^3^-phenyl); 7.20–7.34 (m, 2H, benzimidazole-C^5,6^-Hs); 7.45 (dist. dd, 1H, C^4^-H of pyrazole N^1^-phenyl); 7.56–7.81 (m, 13H, C^2,3,5,6^-Hs of thiazolidinone N^3^-phenyl, -C = CH vinylic, benzimidazole C^4,7^-Hs, C^2,3,5,6^-Hs of pyrazole N^1^-phenyl and C^3,5^-Hs of p-Cl-phenyl ring); 8.08 (d, J = 7.5 Hz, 2H, C^2,6^-Hs of p-Cl-phenyl ring); 8.94 (s, 1H, pyrazole C^5^-H); 11.21 (s, 1H, -NH of benzimidazole, D_2_O exchangeable). Anal. Calcd. for C_34_H_24_ClN_7_OS: C, 66.50; H, 3.94; N, 15.97. Found: C, 66.48; H, 3.98; N, 16.13.

##### 2-[1-(1H-Benzimidazole-2-yl)ethylidenehydrazono]-5-{[3-(4-methoxyphenyl)-1-phenyl-1H-pyrazol-4-yl]methylene}-3-phenylthiazolidin-4-one 5o

Yellow solid, yield 56%, m.p. >300°C. FT-IR (cm^-1^): 3319 (N-H); 1719 (C = O); 1617 (C = N). EIMS m/z (% abundance), 611 (14.83) M^+^ + 2; 610 (42.72) M^+^ + 1; 609 (100) M^+^. Anal. Calcd. for C_35_H_27_N_7_O_2_S: C, 68.95; H, 4.46; N, 16.08. Found: C, 69.07; H, 4.53; N, 16.22.

##### 2-[1-(1H-Benzimidazole-2-yl)ethylidenehydrazono]-5-[(1,3-Diphenyl-1H-pyrazol-4-yl)methylene]-3-(4-chlorophenyl)thiazolidin-4-one 5p

Yellow solid, yield 72%, m.p. 286–8. FT-IR (cm^-1^): 3319 (N-H); 1723 (C = O); 1598 (C = N). ^1^H-NMR (DMSO-d_6_, Jeol 500 MHz): δ 2.33 (s, 3H, -N = C-CH_3_); 7.18–7.23 (dist. dd, 1H, benzimidazole C^5^-H); 7.28–7.33 (dist. dd, 1H, benzimidazole C^6^-H); 7.45 (t, J = 7.5 Hz, 1H, C^4^-H of pyrazole C^3^-phenyl); 7.49–7.71 (m, 13H, benzimidazole C^4,7^-Hs, C^2,3,5,6^-Hs of p-Cl-phenyl ring, C^2,3,4,5,6^-Hs of pyrazole N^1^-phenyl and C^3,5^-Hs of pyrazole C^3^-phenyl); 7.77 (s, 1H, -C = CH vinylic); 8.06 (d, J = 8 Hz, 2H, C^2,6^-Hs of pyrazole C^3^-phenyl); 8.86 (s, 1H, pyrazole C^5^-H); 12.6 (s, 1H, -NH of benzimidazole, D_2_O exchangeable). Anal. Calcd. for C_34_H_24_ClN_7_OS: C, 66.50; H, 3.94; N, 15.97. Found: C, 66.59; H, 4.02; N, 16.13.

##### 2-[1-(1H-Benzimidazole-2-yl)ethylidenehydrazono]-5-{[3-(4-chlorophenyl)-1-phenyl-1H-pyrazol-4-yl]methylene}-3-(4-chlorophenyl)thiazolidin-4-one 5q

Yellow solid, yield 60%, m.p. >300. FT-IR (cm^-1^): 3335 (N-H); 1715 (C = O); 1603 (C = N). ^1^H-NMR (DMSO-d_6_, Mercury 300 MHz): δ 2.37, 2.58 (2s, 3H, -N = C-CH_3_ due to isomerism); 7.10–7.55 (m, 2H, benzimidazole C^5,6^-H due to isomerism); 7.56–7.95 (m, 14H, C^4,7^-H of benzimidazole, -C = CH vinylic, C^2,3,4,5,6^-Hs of pyrazole N^1^-phenyl, C^2,3,5,6^-Hs of p-Cl-phenyl ring of thiazolidinone and C^3,5^-Hs of p-Cl-phenyl ring of pyrazole); 8.04–8.13 (m, 2H, C^2,6^-Hs of p-Cl-phenyl ring of pyrazole due to isomerism); 8.89, 8.96 (2s, 1H, pyrazole C^5^-H due to isomerism); 11.27, 12.56 (2s, 1H, -NH of benzimidazole, D_2_O exchangeable due to isomerism). Anal. Calcd. for C_34_H_23_Cl_2_N_7_OS: C, 62.96; H, 3.57; N, 15.12. Found: C, 63.09; H, 3.54; N, 15.27.

##### 2-[1-(1H-Benzimidazole-2-yl)ethylidenehydrazono]-5-{[3-(4-methoxyphenyl)-1-phenyl-1H-pyrazol-4-yl]methylene}-3-(4-chlorophenyl)thiazolidin-4-one 5r

Yellow solid, yield 69%, m.p. >300. FT-IR (cm^-1^): 3340 (N-H); 1707 (C = O); 1608 (C = N). ^1^H-NMR (DMSO-d_6_, Mercury 300 MHz): δ 2.37 (s, 3H, -N = C-CH_3_); 3.84 (s, 3H, O-CH_3_); 7.14 (d, J = 8.5 Hz, 2H, C^3,5^-Hs of p-OCH_3_-phenyl ring); 7.20–7.28 (dist. dd, 1H, benzimidazole C^5^-H); 7.30–7.38 (dist. dd, 1H, benzimidazole C^6^-H); 7.46–7.50 (dist. t, 1H, C^4^-H of pyrazole N^1^-phenyl); 7.61–7.68 (m, 9H, benzimidazole C^4^-H, C^2,3,5,6^-Hs of pyrazole N^1^-phenyl and C^2,3,5,6^-Hs of p-Cl-phenyl ring); 7.72 (d, J = 8 Hz, 1H, benzimidazole C^7^-H); 7.80 (s, 1H, -C = CH vinylic); 8.07 (d, J = 8.5 Hz, 2H, C^2,6^-Hs of p-OCH_3_-phenyl ring); 8.94 (s, 1H, pyrazole C^5^-H); 12.61 (s, 1H, -NH of benzimidazole, D_2_O exchangeable). Anal. Calcd. for C_35_H_26_ClN_7_O_2_S.1/2H_2_O: N, 15.01; S, 4.91. Found: N, 14.67; S, 5.23.

##### 2-[1-(1H-Benzimidazole-2-yl)ethylidenehydrazono]-5-[(1,3-Diphenyl-1H-pyrazol-4-yl)methylene]-3-(4-methoxyphenyl)thiazolidin-4-one 5s

Yellow solid, yield 72%, m.p. >300. FT-IR (cm^-1^): 3342 (N-H); 1715 (C = O); 1613 (C = N). ^1^H-NMR (DMSO-d_6_, Mercury 300 MHz): δ 2.57 (s, 3H, -N = C-CH_3_); 3.92 (s, 3H, -OCH_3_); 7.02–7.52 (m, 5H, C^3,5^-Hs of p-OCH_3_-phenyl ring, benzimidazole C^5,6^-H and C^4^-H of pyrazole C^3^-phenyl); 7.53–7.77 (m, 12H, C^2,6^-Hs of p-OCH_3_-phenyl ring, benzimidazole C^4,7^-H, -C = CH vinylic, C^2,3,4,5,6^-Hs of pyrazole N^1^-phenyl and C^3,5^-Hs of pyrazole C^3^-phenyl); 8.06–8.14 (dist. d, 2H, C^2,6^-Hs of pyrazole C^3^-phenyl); 8.95 (br. s, 1H, pyrazole C^5^-H); 11.27 (s, 1H, -NH of benzimidazole, D_2_O exchangeable). Anal. Calcd. for C_35_H_27_N_7_O_2_S: C, 68.95; H, 4.46; N, 16.08; S, 5.26. Found: C, 68.74; H, 4.29; N, 16.07; S, 5.51.

##### 2-[1-(1H-Benzimidazole-2-yl)ethylidenehydrazono]-5-{[3-(4-chlorophenyl)-1-phenyl-1H-pyrazol-4-yl]methylene}-3-(4-methoxyphenyl)thiazolidin-4-one 5t

Yellow solid, yield 72%, m.p. >300. FT-IR (cm^-1^): 3327 (N-H); 1707 (C = O); 1597 (C = N). ^1^H-NMR (DMSO-d_6_, Mercury 300 MHz): δ 2.37 (s, 3H, -N = C-CH_3_); 3.93 (s, 3H, -OCH_3_); 7.06–7.78 (m, 16H, benzimidazole C^4,5,6,7^-Hs, C^2,3,5,6^-Hs of p-OCH_3_-phenyl ring, C^2,3,4,5,6^-Hs of pyrazole N^1^-phenyl, C^3,5^-Hs of p-Cl-phenyl ring and -C = CH vinylic); 8.06–8.13 (dist. d, 2H, C^2,6^-Hs of p-Cl-phenyl ring); 8.99 (br. s, 1H, pyrazole C^5^-H); 11.28 (s, 1H, -NH of benzimidazole, D_2_O exchangeable). Anal. Calcd. for C_35_H_26_ClN_7_O_2_S: C, 65.26; H, 4.07; N, 15.22. Found: C, 65.41; H, 4.13; N, 15.43.

##### 2-[1-(1H-Benzimidazole-2-yl)ethylidenehydrazono]-5-{[3-(4-methoxyphenyl)-1-phenyl-1H-pyrazol-4-yl]methylene}-3-(4-methoxyphenyl)thiazolidin-4-one 5u

Yellow solid, yield 79%, m.p. >300. FT-IR (cm^-1^): 3341 (N-H); 1712 (C = O); 1600 (C = N). ^1^H-NMR (DMSO-d_6_, Jeol 500 MHz): δ 2.55 (s, 3H, -N = C-CH_3_); 3.81 (s, 3H, O-CH_3_ of p-OCH_3_-phenyl of thiazolidinone); 3.90 (s, 3H, O-CH_3_ of p-OCH_3_-phenyl ring of pyrazole); 7.00–7.16 (dist. 2d, 4H, C^3,5^-Hs of p-OCH_3_-phenyl ring of thiazolidinone and C^3,5^-Hs of p-OCH_3_-phenyl ring of pyrazole); 7.22–7.32 (m, 3H, C^2,6^-Hs of p-OCH_3_-phenyl ring of thiazolidinone and benzimidazole C^5^-H); 7.37–7.44 (dist. dd, 1H, benzimidazole C^6^-H); 7.54–7.72 (m, 8H, C^3,4,5^-Hs of pyrazole N^1^-phenyl, C^4,7^-H of benzimidazole, C^2,6^-Hs of p-OCH_3_-phenyl ring of pyrazole, -C = CH vinylic); 8.00–8.07 (m, 2H, C^2,6^-Hs of pyrazole N^1^-phenyl); 8.98 (br. s, 1H, pyrazole C^5^-H); 11.25 (s, 1H, -NH of benzimidazole, D_2_O exchangeable). Anal. Calcd. for C_36_H_29_N_7_O_3_S.1/2H_2_O: C, 66.65; H, 4.66; N, 15.11; S, 4.94. Found: C, 66.49; H, 4.63; N, 15.02; S, 5.02.

##### 2-[1-(1H-Benzimidazole-2-yl)ethylidenehydrazono]-5-[(1,3-Diphenyl-1H-pyrazol-4-yl)methylene]-3-cyclohexylthiazolidin-4-one 5v

Yellow solid, yield 71%, m.p. 230–2. FT-IR (cm^-1^): 3320 (N-H); 1700 (C = O); 1610 (C = N). ^1^H-NMR (DMSO-d_6_, Jeol 500 MHz): δ 1.03–1.40, 1.61–1.86 and 2.35–2.42 (3m, 10H, cyclohexyl C^2,3,4,5,6^-H_2_); 2.59 (s, 3H, -N = C-CH_3_); 4.43–4.51 (m, 1H, cyclohexyl C^1^-H); 7.18–7.24 (dist. dd, 1H, benzimidazole C^5^-H); 7.27–7.34 (dist. dd, 1H, benzimidazole C^6^-H); 7.44 (t, J = 7.5 Hz, 1H, C^4^-H of pyrazole C^3^-phenyl); 7.47–7.67 (m, 9H, -C = CH vinylic, C^3,5^-Hs of pyrazole C^3^-phenyl, benzimidazole C^4^-H and C^2,3,4,5,6^-Hs of pyrazole N^1^-phenyl); 7.70 (d, J = 8 Hz, 1H, benzimidazole C^7^-H); 8.02 (d, J = 8 Hz, 2H, C^2,6^-Hs of pyrazole C^3^-phenyl); 8.79 (s, 1H, pyrazole C^5^-H); 12.56 (s, 1H, -NH of benzimidazole, D_2_O exchangeable). Anal. Calcd. for C_34_H_31_N_7_OS.1/2H_2_O: C, 68.66; H, 5.42; N, 16.49; S, 5.39. Found: C, 68.21; H, 5.34; N, 16.16; S, 5.52.

##### 2-[1-(1H-Benzimidazole-2-yl)ethylidenehydrazono]-5-{[3-(4-chlorophenyl)-1-phenyl-1H-pyrazol-4-yl]methylene}-3-cyclohexylthiazolidin-4-one 5w

Yellow solid, yield 82%, m.p. 167–9. FT-IR (cm^-1^): 3336 (N-H); 1714 (C = O); 1611 (C = N). ^1^H-NMR (DMSO-d_6_, Mercury 300 MHz): δ 1.11–1.48, 1.64–1.90 and 2.32–2.49 (3m, 10H, cyclohexyl C^2,3,4,5,6^-H_2_); 2.61 (s, 3H, -N = C-CH_3_); 4.47–4.56 (m, 1H, cyclohexyl C^1^-H); 7.20–7.28 (dist. dd, 1H, benzimidazole C^5^-H); 7.30–7.37 (dist. dd, 1H, benzimidazole C^6^-H); 7.47 (t, J = 7.5 Hz, 1H, C^4^-H of pyrazole N^1^-phenyl); 7.52 (s, 1H, -C = CH vinylic); 7.58–7.67 (m, 5H, C^3,5^-Hs of p-Cl-phenyl ring, C^3,5^-Hs of pyrazole N^1^-phenyl, benzimidazole C^4^-H); 7.68–7.76 (m, 3H, C^2,6^-Hs of pyrazole N^1^-phenyl and benzimidazole C^7^-H); 8.04 (d, J = 8 Hz, 2H, C^2,6^-Hs of p-Cl-phenyl ring); 8.81 (s, 1H, pyrazole C^5^-H); 12.52 (s, 1H, -NH of benzimidazole, D_2_O exchangeable). Anal. Calcd. for C_34_H_30_ClN_7_OS: C, 65.85; H, 4.88; N, 15.81. Found: C, 65.98; H, 4.96; N, 16.02.

##### 2-[1-(1H-Benzimidazole-2-yl)ethylidenehydrazono]-5-{[3-(4-methoxyphenyl)-1-phenyl-1H-pyrazol-4-yl]methylene}-3-cyclohexylthiazolidin-4-one 5x

Yellow solid, yield 68%, m.p. 215–7. FT-IR (cm^-1^): 3337 (N-H); 1720 (C = O); 1596 (C = N). ^1^H-NMR (DMSO-d_6_, Mercury 300 MHz): δ 1.11–1.47 and 1.64–1.88 (2m, 10H, cyclohexyl C^2,3,4,5,6^-H_2_); 2.60 (s, 3H, -N = C-CH_3_); 3.82 (s, 3H, O-CH_3_); 4.44–4.52 (m, 1H, cyclohexyl C^1^-H); 7.12 (d, J = 7.5 Hz, 2H, C^3,5^-Hs of p-OCH_3_-phenyl ring); 7.19–7.26 (dist. dd, 1H, benzimidazole C^5^-H); 7.28–7.35 (dist. dd, 1H, benzimidazole C^6^-H); 7.40–7.48 (m, 1H, C^4^-H of pyrazole N^1^-phenyl); 7.51 (s, 1H, -C = CH vinylic); 7.56–7.64 (m, 5H, benzimidazole C^4^-H, C^2,3,5,6^-Hs of pyrazole N^1^-phenyl); 7.68–7.74 (dist. d, 1H, benzimidazole C^7^-H); 8.02 (d, J = 7.5 Hz, 2H, C^2,6^-Hs of p-OCH_3_ phenyl ring); 8.77 (s, 1H, pyrazole C^5^-H); 12.56 (s, 1H, -NH of benzimidazole, D_2_O exchangeable). Anal. Calcd. for C_35_H_33_N_7_O_2_S.1/2H_2_O: C, 67.29; H, 5.49; N, 15.69; S, 5.13. Found: C, 66.89; H, 5.25; N, 15.50; S, 4.94.

#### General procedure for the synthesis of compounds 6a-h

To a mixture of 2-[1-(benzofuran-2-yl)ethylidenehydrazono]-3-substituted thiazolidin-4-ones or 2-[1-(1*H*-benzimidazol-2-yl)ethylidenehydrazono]-3-substituted thiazolidin-4-ones **(3a-i)** (1 mmol) and isatin (0.16 g, 1.1 mmol) in dry dioxane (5 ml), two drops of piperidine were added. The reaction mixture was refluxed for 4–6 h, concentrated, then allowed to cool to room temperature. The red precipitate formed after addition of ethanol (10 ml) was filtered, washed with ethanol and recrystallized from dioxane/ethanol.

##### 2-[1-(Benzofuran-2-yl)ethylidenehydrazono]-5-(2-oxoindolin-3-ylidene)-3-phenylthiazolidin-4-one 6a

Red solid, yield 63%, m.p. >300. FT-IR (cm^-1^): 3193 (N-H); 1705 (C = O of thiazolidinone); 1687 (amide I band of isatin); 1606 (C = N); 1263, 1174, 1082 (C-O-C). ^1^H-NMR (DMSO-d_6_, Mercury 300 MHz): δ 2.24 (s, 3H, -N = C-CH_3_); 6.96 (d, J = 8 Hz, 1H, isatin C^7^-H); 6.99–7.07 (dist. dd, 1H, isatin C^5^-H); 7.27–7.7.61 (m, 9H, benzofuran C^3,5,6^-Hs, Ar-C^2,3,4,5,6^-Hs and isatin C^6^-H); 7.72–7.77 (m, 2H, benzofuran C^4,7^-Hs); 8.79 (d, J = 8 Hz, 1H, isatin C^4^-H); 11.21 (s, 1H, -NH of isatin, D_2_O exchangeable). Anal. Calcd. for C_27_H_18_N_4_O_3_S: C, 67.77; H, 3.79; N, 11.71. Found: C, 67.91; H, 3.84; N, 11.87.

##### 2-[1-(Benzofuran-2-yl)ethylidenehydrazono]-5-(2-oxoindolin-3-ylidene)-3-(4-chlorophenyl)thiazolidin-4-one 6b

Red solid, yield 65%, m.p. >300. FT-IR (cm^-1^): 3189 (N-H); 1703 (C = O of thiazolidinone); 1686 (amide I band of isatin); 1602 (C = N); 1267, 1170, 1087 (C-O-C). ^1^H-NMR (DMSO-d_6_, Mercury 300 MHz): δ 2.26 (s, 3H, -N = C-CH_3_); 6.96 (d, J = 7.5 Hz, 1H, isatin C^7^-H); 6.99–7.08 (dist. dd, 1H, isatin C^5^-H); 7.27–7.48 (m, 3H, benzofuran C^5,6^-Hs and isatin C^6^-H); 7.56 (s, 1H, benzofuran C^3^-H); 7.62–7.70 (m, 4H, Ar-C^2,3,5,6^-Hs); 7.71–7.78 (m, 2H, benzofuran C^4,7^-Hs); 11.22 (s, 1H, -NH of isatin, D_2_O exchangeable). Anal. Calcd. for C_27_H_17_ClN_4_O_3_S: C, 63.22; H, 3.34; N, 10.92. Found: C, 63.34; H, 3.39; N, 11.13.

##### 2-[1-(Benzofuran-2-yl)ethylidenehydrazono]-5-(2-oxoindolin-3-ylidene)-3-(4-methoxyphenyl)thiazolidin-4-one 6c

Red solid, yield 78%, m.p. >300. FT-IR (cm^-1^): 3180 (N-H); 1700 (C = O of thiazolidinone); 1683 (amide I band of isatin); 1599 (C = N); 1277, 1167, 1082 (C-O-C). ^1^H-NMR (DMSO-d_6_, Mercury 300 MHz): δ 2.26 (s, 3H, -N = C-CH_3_); 3.84 (s, 3H, O-CH_3_); 6.95 (d, J = 8 Hz, 1H, isatin C^7^-H); 6.99–7.06 (dist. dd, 1H, isatin C^5^-H); 7.12 (d, J = 9 Hz, 2H, Ar-C^3,5^-Hs); 7.28–7.45 (m, 3H, isatin C^6^-H and benzofuran C^5,6^-Hs); 7.50 (d, J = 9 Hz, 2H, Ar-C^2,6^-Hs); 7.55 (s, 1H, benzofuran C^3^-H); 7.72–7.78 (m, 2H, benzofuran C^4,7^-H); 8.80 (d, J = 8 Hz, 1H, isatin C^4^-H); 11.22 (s, 1H, -NH of isatin, D_2_O exchangeable). Anal. Calcd. for C_28_H_20_N_4_O_4_S: C, 66.13; H, 3.96; N, 11.02; S, 6.31. Found: C, 65.77; H, 3.85; N, 11.01; S, 6.68.

##### 2-[1-(Benzofuran-2-yl)ethylidenehydrazono]-5-(2-oxoindolin-3-ylidene)-3-cyclohexylthiazolidin-4-one 6d

Red solid, yield 73%, m.p. >300. FT-IR (cm^-1^): 3166 (N-H); 1707 (C = O of thiazolidinone); 1690 (amide I band of isatin); 1597 (C = N); 1283, 1175, 1080 (C-O-C). ^1^H-NMR (DMSO-d_6_, Mercury 300 MHz): δ 1.17–1.48, 1.66–1.92 and 2.34–2.46 (3m, 10H, cyclohexyl C^2,3,4,5,6^-H_2_); 2.51 (s, 3H, -N = C-CH_3_); 4.50–4.63 (m, 1H, cyclohexyl C^1^-H); 6.94 (d, J = 7.7 Hz, 1H, isatin C^7^-H); 7.02–7.10 (dist. dd, 1H, isatin C^5^-H); 7.29–7.39 (m, 2H, benzofuran C^5,6^-Hs); 7.40–7.48 (dist. dd, 1H, isatin C^6^-H); 7.60 (s, 1H, benzofuran C^3^-H); 7.76 (m, 2H, benzofuran C^4,7^-Hs); 8.87 (d, J = 7.7 Hz, 1H, isatin C^4^-H); 11.15 (s, 1H, -NH of isatin, D_2_O exchangeable). Anal. Calcd. for C_27_H_24_N_4_O_3_S: C, 66.92; H, 4.99; N, 11.56. Found: C, 67.09; H, 5.07; N, 11.73.

##### 2-[1-(1H-Benzimidazole-2-yl)ethylidenehydrazono]-5-(2-oxoindolin-3-ylidene)-3- phenylthiazolidin-4-one 6e

Red solid, yield 60%, m.p. >300. FT-IR (cm^-1^): 3257, 3119 (N-H); 1704 (C = O of thiazolidinone); 1680 (amide I band of isatin); 1607 (C = N). ^1^H-NMR (DMSO-d_6_, Mercury 300 MHz): δ 2.33 (s, 3H, -N = C-CH_3_); 6.97 (d, J = 7.5 Hz, 1H, isatin C^7^-H); 6.99–7.06 (dist. dd, 1H, isatin C^5^-H); 7.16–7.24 (dist. dd, 1H, benzimidazole C^5^-H); 7.26–7.40 (m, 2H, benzimidazole C^6^-H and isatin C^6^-H); 7.51–7.62 (m, 6H, benzimidazole C^4^-H and Ar-C^2,3,4,5,6^-Hs); 7.69 (d, J = 8 Hz, 1H, benzimidazole C^7^-H); 8.78 (d, J = 7.5 Hz, 1H, isatin C^4^-H); 11.31, 12.76 (2s, each 1H, -NH of isatin and benzimidazole, D_2_O exchangeable). Anal. Calcd. for C_26_H_18_N_6_O_2_S: C, 65.26; H, 3.79; N, 17.56. Found: C, 65.41; H, 3.85; N, 17.75.

##### 2-[1-(1H-Benzimidazole-2-yl)ethylidenehydrazono]-5-(2-oxoindolin-3-ylidene)-3-(4-chlorophenyl)thiazolidin-4-one 6f

Red solid, yield 70%, m.p. >300. FT-IR (cm^-1^): 3260, 3123 (N-H); 1714 (C = O of thiazolidinone); 1682 (amide I band of isatin); 1610 (C = N). ^1^H-NMR (DMSO-d_6_, Mercury 300 MHz): δ 2.35 (s, 3H, -N = C-CH_3_); 6.96 (d, J = 8 Hz, 1H, isatin C^7^-H); 6.99–7.07 (dist. dd, 1H, isatin C^5^-H); 7.16–7.40 (m, 3H, benzimidazole C^5,6^-Hs and isatin C^6^-H); 7.58–7.74 (m, 6H, Ar-C^2,3,5,6^-Hs and benzimidazole C^4,7^-Hs); 8.79 (d, J = 8 Hz, 1H, isatin C^4^-H); 11.23, 12.72 (2s, each 1H, -NH of isatin and benzimidazole, D_2_O exchangeable). EIMS m/z (% abundance), 513 (31.40) M^+^ + 1; 512 (42.72) M^+^; 64 (100). Anal. Calcd. for C_26_H_17_ClN_6_O_2_S: C, 60.88; H, 3.34; N, 16.38. Found: C, 60.83; H, 3.41; N, 16.49.

##### 2-[1-(1H-Benzimidazole-2-yl)ethylidenehydrazono]-5-(2-oxoindolin-3-ylidene)-3-(4-methoxyphenyl)thiazolidin-4-one 6g

Red solid, yield 65%, m.p. >300. FT-IR (cm^-1^): 3267, 3133 (N-H); 1708 (C = O of thiazolidinone); 1684 (amide I band of isatin); 1611 (C = N). ^1^H-NMR (DMSO-d_6_, Jeol 500 MHz): δ 2.30 (s, 3H, -N = C-CH_3_); 3.81 (s, 3H, O-CH_3_); 6.92 (d, J = 7.5 Hz, 1H, isatin C^7^-H); 6.98–7.04 (dist. dd, 1H, isatin C^5^-H); 7.09 (d, J = 9 Hz, 2H, Ar-C^3,5^-Hs); 7.16–7.22 (dist. dd, 1H, benzimidazole C^5^-H); 7.25–7.30 (dist. dd, 1H, benzimidazole C^6^-H); 7.30–7.36 (dist. dd, 1H, isatin C^6^-H); 7.47 (d, J = 9 Hz, 2H, Ar-C^2,6^-Hs); 7.58 (d, J = 8 Hz, 1H, benzimidazole C^4^-H); 7.66 (d, J = 8 Hz, 1H, benzimidazole C^7^-H); 8.78 (d, J = 7.5 Hz, 1H, isatin C^4^-H); 11.24, 12.73 (2s, each 1H, -NH of isatin and benzimidazole, D_2_O exchangeable). Anal. Calcd. for C_27_H_20_N_6_O_3_S: C, 63.77; H, 3.96; N, 16.53. Found: C, 63.85; H, 4.03; N, 16.71.

##### 2-[1-(1H-Benzimidazole-2-yl)ethylidenehydrazono]-5-(2-oxoindolin-3-ylidene)-3-cyclohexylthiazolidin-4-one 6h

Red solid, yield 56%, m.p. >300. FT-IR (cm^-1^): 3263, 3137 (N-H); 1715 (C = O of thiazolidinone); 1680 (amide I band of isatin); 1602 (C = N). ^1^H-NMR (DMSO-d_6_, Jeol 500 MHz): δ 1.09–1.41, 1.60–1.90 and 2.29–2.42 (3m, 10H, cyclohexyl C^2,3,4,5,6^-H_2_); 2.58 (s, 3H, -N = C-CH_3_); 4.51- 4.61 (m, 1H, cyclohexyl C^1^-H); 6.91 (d, J = 8 Hz, 1H, isatin C^7^-H); 7.02–7.08 (dist. dd, isatin C^5^-H); 7.17–7.23 (dist. dd, 1H, benzimidazole C^5^-H); 7.25–7.31 (dist. dd, 1H, isatin C^6^-H); 7.31–7.37 (dist. dd, 1H, benzimidazole C^6^-H); 7.59 (d, J = 8 Hz, 1H, benzimidazole C^4^-H); 7.69 (d, J = 8 Hz, 1H, benzimidazole C^7^-H); 8.86 (d, J = 8 Hz, 1H, isatin C^4^-H); 11.18, 12.73 (2s, each 1H, -NH of isatin and benzimidazole, D_2_O exchangeable). Anal. Calcd. for C_26_H_24_N_6_O_2_S.1/2H_2_O: C, 63.27; H, 5.11; N, 17.03; S, 6.50. Found: C, 63.23; H, 5.04; N, 16.67; S, 6.35.

#### Synthesis of 2-{[3-(4-chlorophenyl)-1-phenyl-1H-pyrazol-4-yl]methylenehydrazono}-3-cyclohexyl-thiazolidin-4-one 7

To a solution of 2-[1-(benzofuran-2-yl)ethylidenehydrazono]-3-cyclohexyl-thiazolidin-4-one or 2-[1-(1*H*-benzimidazol-2-yl)ethylidenehydrazono]-3-cyclohexyl-thiazolidin-4-one **(3b or 3i)** (0.355 g, 1 mmol) and 3-(4-chlorophenyl)-1-phenyl-1*H*-pyrazole-4-carbaldehyde **(4b)** (0.31 g, 1.1 mmol) in glacial acetic acid (5 ml), anhydrous sodium acetate (0.13 g, 1.5 mmol) was added. The reaction mixture was refluxed for 10 h, then filtered while hot and the solid separated was filtered, washed with ethanol and recrystallized from dioxane/ethanol.

Yellow solid, yield 65%, m.p. >300. FT-IR (cm^-1^): 1694 (C = O); 1618 (C = N). ^1^H-NMR (DMSO-d_6_, Jeol 500 MHz): δ 1.11–1.27, 1.55–1.78 and 2.25–2.28 (3m, 10H, cyclohexyl C^2,3,4,5,6^-H_2_); 3.89 (s, 2H, thiazolidinone C^5^-H_2_); 4.20–4.32 (m, 1H, cyclohexyl C^1^-H); 7.33–7.41 (m, 1H, C^4^-H of pyrazole N^1^-phenyl); 7.48–7.63 (m, 4H, C^3,5^-Hs of pyrazole N^1^-phenyl and C^3,5^-Hs of p-Cl-phenyl ring); 7.91 (d, J = 7.5 Hz, 2H, C^2,6^-Hs of pyrazole N^1^-phenyl); 7.97 (d, J = 7 Hz, 2H, C^2,6^-Hs of p-Cl-phenyl ring); 8.50 (s, 1H, HC = N-); 8.96 (s, 1H, pyrazole C^5^-H). Anal. Calcd. for C_25_H_24_ClN_5_OS.1/2H_2_O: C, 61.65; H, 5.17; N, 14.38; S, 6.58. Found: C, 62.00; H, 5.30; N, 14.21; S, 6.21.

#### Synthesis of 3-cyclohexyl-5-(2-oxoindolin-3-ylidene)-2-[(2-oxoindolin-3-ylidene)hydrazono]thiazolidin-4-one 8

To a solution of 2-[1-(3-cyclohexyl-4-oxothiazolidin-2-ylidene)hydrazonoethyl]benzofuran (**3d**) (0.355 g, 1 mmol) and isatin (0.16 g, 1.1 mmol) in glacial acetic acid (5 ml), anhydrous sodium acetate (0.13 g, 1.5 mmol) was added. The reaction mixture was refluxed for 4 h, then filtered while hot and the solid separated was filtered, washed with ethanol and recrystallized from dioxane/ethanol.

Red solid, yield 38%, m.p. >300. FT-IR (cm^-1^): 3160 (N-H); 1725 (C = O of thiazolidinone); 1693 (amide I band of isatin); 1608 (C = N). ^1^H-NMR (DMSO-d_6_, Jeol 500 MHz): δ 1.07–1.49 and 1.56–1.94 (2m, 10H, cyclohexyl C^2,3,4,5,6^-H_2_); 4.42–4.70 (m, 1H, cyclohexyl C^1^-H); 6.80–6.93 (m, 2H, two isatin C^7^-Hs); 6.96–7.08 (m, 2H, two isatin C^5^-Hs); 7.27–7.41 (m, 2H, two isatin C^6^-Hs); 8.15 and 8.80 (2 d, J = 7 and 7.5 Hz, each 1H, 2 isatin C^4^-Hs); 10.79 and 11.18 (2 s, each 1H, 2 -NHs, D_2_O exchangeable). Anal. Calcd. for C_25_H_21_N_5_O_3_S: C, 63.68; H, 4.49; N, 14.85. Found: C, 63.81; H, 4.54; N, 15.02.

### Biological evaluation

#### *In vitro* hemolytic assay

Blood samples (5 ml) were freshly collected into heparinized tubes and were centrifuged (1000 r.p.m. for 20 min) at room temperature. The plasma and buffy coat were removed by Pasteur pipette, and the collected erythrocytes were washed three-times using isotonic buffer solution (0.9% sodium chloride [Sigma-Aldrich, MO, USA]) by centrifugation (2500 r.p.m. for 10 min). A two percent erythrocyte suspension was prepared to be used in the assay.

A 1 mg/ml stock solution of each compound was prepared in DMF (Sigma-Aldrich). Compound wells were prepared by adding 3 μl of the compound solution to 117 μl isotonic buffer solution and 120 μl erythrocyte suspension. Compounds color blank wells were prepared by adding 3 μl compound to 237 μl isotonic buffer solution. Solvent (DMF) wells were prepared by adding 3 μl DMF to 117 μl isotonic buffer solution and 120 μl erythrocyte suspension. Positive control wells (representing 100% hemolysis) were prepared by adding 120 μl erythrocyte suspension to 120 μl distilled water, while negative control wells (representing 0% hemolysis) were prepared by adding 120 μl erythrocyte suspension to 120 μl isotonic buffer solution. Finally, blank wells contained only 240 μl isotonic buffer solution. Each set of samples was pipetted in triplicate. The microtiter plate was then shaken well on bench, incubated at 37°C for 30 min then centrifuged (2000 rpm for 5 min) at room temperature.

A fraction of the supernatant layer (150 μl) of each well was transferred to another 96-well microtiter plate. Erythrocyte hemolysis was determined by reading the absorbance (**A**) of liberated hemoglobin at 405 nm in the supernatant fraction compared with the 100% hemolysis of erythrocyte. The average value was calculated from triplicate assay:
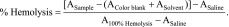



It was decided during the present investigation that compounds which caused <5% hemolysis were considered nontoxic to RBCs, while those which demonstrated 5% or more hemolysis were considered toxic and hemolytic on RBCs.

#### *In vitro* anticancer screening

HepG2 cells were routinely maintained as adherent cell cultures in RPMI-1640 medium (Lonza Group Ltd, Basel, Switzerland) supplemented with 10% fetal bovine serum (FBS; Lonza, IL, USA) at 37°C in a humidified air incubator containing 5% CO_2_. Cells were subcultured for 2 weeks before assay. Cell viability was assessed using trypan blue exclusion method.

HepG2 cells were washed twice in RPMI-1640 medium supplemented with 200 μM L-glutamine (Lonza) and 25 μM 4-(2-hydroxyethyl)-1-piperazineethanesulfonic acid (HEPES) buffer (Lonza). The cells were suspended at 3 × 10^4^ cells/ml in RPMI culture medium (RPMI supplemented medium and 10% FBS). The appropriate number of cells was chosen to be 3 × 10^3^ cells/well (100 μl of the prepared suspension), and the cells were left to adhere on the polystyrene 96-well plates in an incubator at 37°C, 5% CO_2_ and 95% humidity for 24 h. The cells were washed once using RPMI supplemented medium.

A 2 mg/ml stock solution of each compound was prepared in DMF and filtered using a 0.2 μM syringe filter. The desired concentrations (20, 10, 5, 2.5 and 1.25 μg/ml) were prepared using serial dilution in a 96-well plate. Compound wells were prepared by adding 100 μl of the previously prepared concentrations to a 100 μl of HepG2 cells suspension. Parallel concentrations of the solvent were prepared to be used as controls. 5-Fluorouracil was used as a positive control. Control wells were prepared by adding 100 μl culture media to a 100 μl of HepG2 cells suspension. Blank wells contained 200 μl of culture media only (without cells or compound solution). Each set of samples was pipetted in duplicate. The plate was gently shaken, then incubated at 37°C, 5% CO_2_ for 72 h.

After incubation, the plate was centrifuged (2000 r.p.m. for 10 min). The media were discarded by inversion over paper towels. A working solution (100 μg/ml) of neutral red stain (Bio Basic Inc, Markham, Canada) was prepared, and 100 μl of this solution was added to each well, then the plate was gently shaken. The plate was incubated at 37°C in humidified 5% CO_2_ for 3 h, and then centrifuged (2000 r.p.m. for 10 min). Excess dyes were discarded, and the cells were fixed with 100 μl fixing solution (0.5% formalin with 1% calcium chloride [Sigma-Aldrich]) for 1 min. Cells were destained in 100 μl destaining solution (50% ethanol with 1% glacial acetic acid [Sigma-Aldrich]) for 5 min by shaking. The stain intensity was assayed using automated microplate reader spectrophotometer adjusted at 490 nm. Surviving cell fraction was calculated according to the following equation:
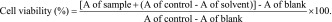



The results were interpreted to calculate both the concentration causing 50% cancer cell death (IC_50_) of each compound and the maximum safe concentration that cause 100% viability (LD_0_; to be used in the *in vitro* anti-HCV testing) using GraphPad InStat 3.0 software [47].

#### *In vitro* cytotoxicity assay

The PBMCs were isolated by density gradient centrifugation technique as described by Boyum [66]. Blood samples were freshly collected into heparinized or ethylenediaminetetraacetic acid sterile tubes. Blood was diluted using equal volume of RPMI-1640 medium containing 25 mM HEPES and then layered over equal volume of Ficoll-Pague™ Plus (Fisher BioReagents™ Lymphocyte Separation Medium-LSM, PA, USA) and centrifuged (2000 r.p.m. for 30 min at acceleration and deceleration speeds zero & zero) at room temperature. The buffy mononuclear cell layer was collected using sterile Pasteur pipette into 50 ml sterile Falcon tube and washed twice in phosphate-buffered saline (Sigma-Aldrich) using centrifugation (1650 r.p.m. for 5 min). The isolated PBMCs viability was determined by hemocytometer count using the trypan blue exclusion method. The PBMCs were resuspended at 1 × 10^6^ cells/ml in RPMI-1640 medium containing 25 mM HEPES supplemented with 10% heat-inactivated FBS. The appropriate number of cells was chosen to be 1 × 10^5^ cells/well (100 μl of the prepared suspension).

A 2 mg/ml stock solution of each compound was prepared in DMF and filtered using a 0.2 μM syringe filter. The desired concentrations (20, 10, 5, 2.5 and 1.25 μg/ml) were prepared using serial dilution in a 96-well plate. Compound wells were prepared by adding 100 μl of the previously prepared concentrations to a suspension of 1 × 10^5^ PBMCs in 100 μl culture media. Parallel concentrations of the solvent were prepared to be used as controls. Control wells were prepared by adding 100 μl culture media to a suspension of 1 × 10^5^ PBMCs in 100 μl culture media. Blank wells contained 200 μl of culture media only (without cells or compound solution). Each set of samples was pipetted in duplicate. The plate was then gently shaken then incubated at 37°C, 5% CO_2_ for 72 h.

After incubation, neutral red assay was performed as described previously, and surviving cell fraction was calculated according to the previously mentioned equation. Also, the results were interpreted to calculate both the lethal concentration that kills 50% of cells (LD_50_) and the maximum safe concentration that cause 100% viability (LD_0_) of each compound using GraphPad InStat 3.0 software [47].

#### *In vitro* preliminary anti-HCV screening

HepG2 cells were washed twice in RPMI-1640 medium supplemented with 200 μM L-glutamine and 25 μM HEPES buffer. The cells were suspended at 12 × 10^4^ cells/ml in RPMI culture medium (RPMI supplemented medium and 10% FBS). The appropriate number of cells to be used was chosen to be 12 × 10^3^ cells/well (100 μl of the prepared suspension), and the cells were left to adhere on the polystyrene 12-well plates in an incubator at 37°C, 5% CO_2_ and 95% humidity for 24 h. The cells were washed once using RPMI supplemented medium, then infected with 2% HCV-infected serum (the used serum was a pole of ten patients infected with HCV genotype-4 with viral loads: 3.2–9 million IU/ml, G4 HCV RNA) in RPMI culture medium containing 8% FBS. The used dose of each compound was chosen so as to maintain 100% viability of the HepG2 cell line (the dose was lower than the LD_0_ of each compound on HepG2 cells as calculated from the *in vitro* anticancer screening). It was reported that, cultured HepG2 cells lost her biotransformation activity due to decrease in cytochrome (CYP) transcription [67]. This waived its effect on the compounds stability during the test. Negative control is HepG2 cells with culture medium without addition of HCV infected serum or addition of tested compounds. Positive control is HepG2 cells with culture medium and infected HCV serum but without addition of tested compounds. After addition of the compounds, the plates were incubated at 37°C, 5% CO_2_ and 95% humidity for 72 h, followed by RNA extraction. The RNA strand was detected by RT-PCR using HCV-specific primers to the 59-untranslated region of the virus.

RNA was extracted from HepG2 cells using the method described by El-Awady* et al*. [50]. Culture cells were mixed with 200 μl of 4 mol/l guanidinium isothiocyanate (Fischer Bioreagents) containing 25 mmol/l sodium citrate (MP Biomedicals, CA, USA), 0.5% sarkosyl (Fischer Bioreagents), 0.1 mol/l β-mercaptoethanol (MP Biomedicals), and 100 μl sodium acetate (Merck). The lysed cells were mixed with an equal volume of phenol, chloroform and isoamyl alcohol (25:24:1, pH 4; Fischer Bioreagents). After vortexing, the mixture was centrifuged (14,000 r.p.m. for 10 min) at 4°C. The aqueous layer was collected and mixed with an equal volume of isopropanol (Acros Organics, Geel, Belgium). After incubation at -20°C overnight, RNA was precipitated by centrifugation (14,000 r.p.m. for 30 min) at 4°C, and the precipitated RNA was washed twice with 70% ethanol.

The complementary DNA and the first PCR reaction of the nested PCR detection system for the HCV-RNA were performed in a 50 μl volume single-step reaction using the Ready-To-Go RT-PCR beads (GE Healthcare, Amersham, UK) and 10 μM from each of the RT downstream primer (1CH), PCR forward primer (2CH) and reverse primer (P2) (Bioneer Corporation, Daejeon, Republic of Korea). The thermal cycling protocol was manipulated as follows: 30 min at 42°C for complementary DNA synthesis, followed by 5 min at 95°C, 35 cycles of 1 min at 94°C, 1 min at 55°C, 1 min at 72°C and final extension 10 min at 72°C.

The nested PCR amplification was performed in 50 μl reaction mixture containing 0.2 mmol from each dNTP (SibEnzyme Ltd., Novosibirsk, Russia), 10 μM from each of the reverse nested primer (D2) and the forward nested primer (F2), two units of taq DNA polymerase (Thermo Fisher Scientific, MA USA) and 10 μl from the RT-PCR reaction product in a 1× buffer. The thermal cycling protocol was manipulated as follows: 5 min at 95°C, 30 cycles of 1 min at 95°C, 45 s at 58°C and 1 min at 72°C and final extension 10 min at 72°C.

Primers’ sequences were as follows; 1CH: 5′-ggtgcacggtctacgagacctc-3′, 2CH: 5′-aactactgtcttcacgcagaa-3′, P2: 5′tgctcatggtgcacggtcta-3′, D2: 5′-actcggctagcagtctcgcg3′ and F2: 5′-gtgcagcctccaggaccc-3′.

The PCR product was mixed with loading buffer by adding 10× bromophenol blue loading buffer to a final concentration of 1×. DNA was separated by agarose gel electrophoresis using 2.5% agarose gel that was impregnated with ethidium bromide (0.5 mg/ml [Sigma-Aldrich]) at 90 V. The electrophoresis was run in standard TBE buffer 1× (54 g of Tris base, 27.5 g of boric acid and 20 ml of 0.5 mol/l ethylenediaminetetraacetic acid [pH 8] in 500 ml of distilled water and a 1× solution is obtained by adding 1 part of the 10× TBE buffer to nine parts of distilled water). During electrophoresis DNA fragments were then visualized using a UV transilluminator, and a fragment of 174 base pairs length was identified in positive samples.

### Molecular modeling

The molecular modeling studies were performed using the Molecular Operating Environment (MOE) [51] software [52]. The 3D structures and conformations of the enzymes were acquired from the PDB website [53].

The targeted compounds were drawn in MOE using the builder module, and collected in a database. The database was prepared by using the option ‘Protonate 3D’ to add hydrogens, calculate partial charges and minimize energy (using Force Field MMFF94x). In addition, the proteins were prepared by deleting the repeating chains, water molecules and any surfactants. Hydrogens were also added to the atoms of the receptor and the partial charges were calculated. The compounds’ database was then docked into the pocket of each protein using the MOE dock. MOE was also used to calculate the best score between the ligands and the enzymes’-binding sites. Scoring was determined as a total of two specific scoring functions: London forces and affinity dG. The resulted database contained the score between the ligands’ conformers and the enzymes’-binding sites in kcal/mol. 30 conformers of each compound were retained with best score by default. The pose that showed the best score (lowest binding energy) was selected to show the ligand–enzyme interactions. To confirm the credibility of our docking results, the pose selection method was adopted to validate our docking protocol [68]. For all the used proteins, their co-crystallized ligands were drawn in MOE, prepared as the targeted compounds (hydrogens, partial charges and energy minimization) and then docked into the active site of the protein using our protocol. The conformer with the best score was superimposed on the original conformation and orientation of the co-crystallized ligand from the co-crystal structure acquired from the (PDB) using PyMOL software [69]. The root-mean-square deviation between the original and docked conformers was calculated by PyMOL and was <1 Å for all the ligands. It was reported that values <1.5 or 2 Å were a sign of a successful and reliable docking protocol [68].

## Conclusion & future perspective

Molecular hybridization is an important concept in drug design based on the combination of pharmacophoric moieties of different bioactive substances to produce a new hybrid compound with improved affinity, selectivity and efficacy and reduced undesired side effects, when compared with the parent drugs. On the other hand, HCV is a contagious liver disease. The disease can be a mild illness lasting a few weeks or a serious, lifelong condition that can lead to liver cirrhosis which can progress to HCC. The HCV protein NS5A is activated by human kinases to produce the active phosphorylated form which upregulates COX-2 expression and promotes the release of matrix metalloprotinase-2 and 9 associated with tumor progression and recurrence in HCC patients. Design and synthesis of hybrid molecules combining common pharmacophores for inhibiting both HCV and HCC will continue to be an objective for the development of new dual inhibitors of HCV and HCC with fewer side effects. In the current research, hybrid compounds were designed to incorporate both anti-HCV and anticancer pharmacophores. The anti-HCV pharmacophore was designed to include either benzofuran or benzimidazole scaffold linked to thiazolidinone moiety. The anticancer pharmacophore was planned to contain moieties like 1,3-diaryl-1*H*-pyrazole or 2-oxoindolin-3-ylidene able to inhibit kinases, specifically tyrosine kinases, to suppress HCC development, angiogenesis and potentiate the anti-HCV activity by inhibiting NS5A activation. Eight compounds exhibited potential *in vitro* anticancer activity against HCC cell line HepG2. Four compounds showed *in vitro* anti-HCV activity against HepG2 cells infected with HCV. As a result, compounds **5l** and **5p** were found to exhibit dual activity against HCV and HCC *in vitro*. Docking studies suggested that the newly synthesized compounds could suppress HCC through VEGFR2 tyrosine kinase inhibition.

**Summary points**

- Design and synthesize novel compounds that exhibit dual activity against hepatitis C virus (HCV) and its associated major complication, hepatocellular carcinoma (HCC) as an alternative to multidrug therapy.
- Hybrid compounds were designed to incorporate both anti-HCV and anticancer pharmacophores. The anti-HCV pharmacophore was designed to encompass different chemical scaffolds such as benzofuran, benzimidazole and thiazolidinone moieties. In addition, the anticancer pharmacophore was planned to contain moieties like 1,3-diaryl-1*H*-pyrazole or 2-oxoindolin-3-ylidene able to inhibit kinases, specifically tyrosine kinases, to suppress HCC development, angiogenesis and potentiate the anti-HCV activity by inhibition of NS5A activation.
- Compounds **5f, 5j, 5l, 5p, 5q, 5r, 6c** and **6d** exhibited potential *in vitro* anticancer activity against HCC cell line HepG2.
- Compounds **5a, 5l, 5p** and **5v** showed *in vitro* anti-HCV activity against HepG2 cells infected with HCV.
- Compounds **5l** and **5p** were found to exhibit dual activity against HCV and HCC *in vitro*.
- Compound **5q** showed a remarkable anticancer effect on HepG2 cancer cells with IC_50_ and IC_100_ values <100 μg/ml and less than its LD_0_ on peripheral blood mononuclear cell normal cells. This compound also showed a very high value of selectivity index indicating high selectivity toward cancerous HepG2 cells. This compound could be considered as a potent, safe and selective anticancer agent against HCC. This compound could be a lead one for further structure modification to achieve more potent anticancer agents.
- Docking studies suggested that the newly synthesized compounds could suppress HCC through VEGFR2 tyrosine kinase inhibition.

## References

[1] Stanaway JD, Flaxman AD, Naghavi M (2016). The global burden of viral hepatitis from 1990 to 2013: findings from the Global Burden of Disease Study 2013. *Lancet*.

[2] Hepatitis C (2017). http://www.who.int/mediacentre/factsheets/fs164/en/.

[3] Manns MP, Buti M, Gane E (2017). Hepatitis C virus infection. *Nat. Rev. Dis. Primers*.

[4] Delemos AS, Chung RT (2014). Hepatitis C treatment: an incipient therapeutic revolution. *Trends Mol. Med.*.

[5] Zhou C, Zhang W, Chen W (2017). Integrated analysis of copy number variations and gene expression profiling in hepatocellular carcinoma. *Sci. Rep.*.

[6] Herman P, Coelho FF (2014). Laparoscopic resection for hepatocellular carcinoma: eastern and western experiences. *Chin. J. Cancer Res.*.

[7] Ghouri YA, Mian I, Rowe JH (2017). Review of hepatocellular carcinoma: epidemiology, etiology, and carcinogenesis. *J. Carcinog.*.

[8] Yamauchi S, Takeuchi K, Chihara K (2015). Hepatitis C virus particle assembly involves phosphorylation of NS5A by the c-Abl tyrosine kinase. *J. Biol. Chem.*.

[9] Nunez O, Fernandez-Martinez A, Majano PL (2004). Increased intrahepatic cyclooxygenase 2, matrix metalloproteinase 2, and matrix metalloproteinase 9 expression is associated with progressive liver disease in chronic hepatitis C virus infection: role of viral core and NS5A proteins. *Gut*.

[10] Macdonald A, Harris M (2004). Hepatitis C virus NS5A: tales of a promiscuous protein. *J. Gen. Virol.*.

[11] Kanda T, Yokosuka O, Omata M (2013). Hepatitis C virus and hepatocellular carcinoma. *Biology*.

[12] Gale M, Kwieciszewski B, Dossett M, Nakao H, Katze MG (1999). Antiapoptotic and oncogenic potentials of hepatitis C virus are linked to interferon resistance by viral repression of the PKR protein kinase. *J. Virol.*.

[13] De Mitri MS, Morsica G, Cassini R (2002). Prevalence of wild-type in NS5A-PKR protein kinase binding domain in HCV-related hepatocellular carcinoma. *J. Hepatol.*.

[14] Huynh H (2010). Molecularly targeted therapy in hepatocellular carcinoma. *Biochem. Pharmacol.*.

[15] Reig M, Torres F, Rodriguez-Lope C (2014). Early dermatologic adverse events predict better outcome in HCC patients treated with sorafenib. *J. Hepatol.*.

[16] Koschny R, Gotthardt D, Koehler C, Jaeger D, Stremmel W, Ganten TM (2013). Diarrhea is a positive outcome predictor for sorafenib treatment of advanced hepatocellular carcinoma. *Oncology*.

[17] Estfan B, Byrne M, Kim R (2013). Sorafenib in advanced hepatocellular carcinoma: hypertension as a potential surrogate marker for efficacy. *Am. J. Clin. Oncol.*.

[18] Gomaa A, Waked I (2017). Management of advanced hepatocellular carcinoma: review of current and potential therapies. *Practice*.

[19] Kudo M (2017). A new era of systemic therapy for hepatocellular carcinoma with regorafenib and lenvatinib. *Liver Cancer*.

[20] Sangro B, Iñarrairaegui M, Bilbao JI (2012). Radioembolization for hepatocellular carcinoma. *J. Hepatol.*.

[21] Aerts M, Benteyn D, Van Vlierberghe H, Thielemans K, Reynaert H (2016). Current status and perspectives of immune-based therapies for hepatocellular carcinoma. *World J. Gastroenterol.*.

[22] Kneteman NM, Howe AYM, Gao T (2009). HCV796: a selective nonstructural protein 5B polymerase inhibitor with potent anti-hepatitis C virus activity *In vitro*, in mice with chimeric human livers, and in humans infected with hepatitis C virus. *Hepatology*.

[23] Chen YL, Zacharias J, Vince R, Geraghty RJ, Wang Z (2012). C-6 aryl substituted 4-quinolone-3-carboxylic acids as inhibitors of hepatitis C virus. *Bioorg. Med. Chem.*.

[24] Vitale G, Corona P, Loriga M (2012). 5-acetyl-2-arylbenzimidazoles as antiviral agents. Part 4. *Eur. J. Med. Chem.*.

[25] Kim ND, Chun H, Park SJ, Yang JW, Kim JW, Ahn SK (2011). Discovery of novel HCV polymerase inhibitors using pharmacophore-based virtual screening. *Bioorg. Med. Chem. Lett.*.

[26] Kaushik-Basu N, Bopda-Waffo A, Talele TT, Basu A, Chen Y, Kucukguzel SG (2008). 4-Thiazolidinones: a novel class of hepatitis C virus NS5B polymerase inhibitors. *Front. Biosci.*.

[27] Rawal RK, Katti SB, Kaushik-Basu N, Arora P, Pan Z (2008). Non-nucleoside inhibitors of the hepatitis C virus NS5B RNA-dependant RNA polymerase: 2-Aryl-3-heteroaryl-1,3-thiazolidin-4-one derivatives. *Bioorg. Med. Chem. Lett.*.

[28] Kammasud N, Boonyarat C, Tsunoda S (2007). Novel inhibitor for fibroblast growth factor receptor tyrosine kinase. *Bioorg. Med. Chem. Lett.*.

[29] Kammasud N, Boonyarat C, Sanphanya K (2009). 5-Substituted pyrido[2,3-d]pyrimidine, an inhibitor against three receptor tyrosine kinases. *Bioorg. Med. Chem. Lett.*.

[30] Kim MH, Tsuhako AL, Co EW (2012). The design, synthesis, and biological evaluation of potent receptor tyrosine kinase inhibitors. *Bioorg. Med. Chem. Lett.*.

[31] Liang G, Liu Z, Wu J, Cai Y, Li X (2012). Anticancer molecules targeting fibroblast growth factor receptors. *Trends Pharmacol. Sci.*.

[32] Thaher BA, Arnsmann M, Totzke F (2011). Tri- and tetrasubstituted pyrazole derivates: regioisomerism switches activity from p38MAP kinase to important cancer kinases. *J. Med. Chem.*.

[33] Strocchi E, Fornari F, Minguzzi M (2012). Design, synthesis and biological evaluation of pyrazole derivatives as potential multi-kinase inhibitors in hepatocellular carcinoma. *Eur. J. Med. Chem.*.

[34] Huang X-F, Lu X, Zhang Y (2012). Synthesis, biological evaluation, and molecular docking studies of N-((1,3-diphenyl-1H-pyrazol-4-yl)methyl)aniline derivatives as novel anticancer agents. *Biorg. Med. Chem.*.

[35] Li X, Lu X, Xing M (2012). Synthesis, biological evaluation, and molecular docking studies of N,1,3-triphenyl-1H-pyrazole-4-carboxamide derivatives as anticancer agents. *Bioorg. Med. Chem. Lett.*.

[36] Zhang J, Yang PL, Gray NS (2009). Targeting cancer with small molecule kinase inhibitors. *Nat. Rev. Cancer*.

[37] Zheng Y, Zheng M, Ling X (2013). Design, synthesis, quantum chemical studies and biological activity evaluation of pyrazole-benzimidazole derivatives as potent Aurora A/B kinase inhibitors. *Bioorg. Med. Chem. Lett.*.

[38] Miyamoto N, Sakai N, Hirayama T (2013). Discovery of N-[5-({2-[(cyclopropylcarbonyl)amino]imidazo[1,2-b]pyridazin-6-yl}oxy)-2-methylph enyl]-1,3-dimethyl-1H-pyrazole-5-carboxamide (TAK-593), a highly potent VEGFR2 kinase inhibitor. *Bioorg. Med. Chem.*.

[39] Rida S, El-Hawash S, Fahmy H, Hazza A, El-Meligy M (2006). Synthesis and *in vitro* evaluation of some novel benzofuran derivatives as potential anti-HIV-1, anticancer, and antimicrobial agents. *Arch. Pharmacal Res.*.

[40] Poyraz M, Sari M, Demirci F, Kosar M, Demirayak S, Büyükgüngör O (2008). Synthesis, crystal structure and biological activity of 1-(1H-benzoimidazol-2-yl)-ethanone thiosemicarbazone and its cobalt complex. *Polyhedron*.

[41] Bondock S, Khalifa W, Fadda AA (2007). Synthesis and antimicrobial evaluation of some new thiazole, thiazolidinone and thiazoline derivatives starting from 1-chloro-3,4-dihydronaphthalene-2-carboxaldehyde. *Eur. J. Med. Chem.*.

[42] Gawande SS, Warangkar SC, Bandgar BP, Khobragade CN (2013). Synthesis of new heterocyclic hybrids based on pyrazole and thiazolidinone scaffolds as potent inhibitors of tyrosinase. *Bioorg. Med. Chem.*.

[43] Omar K, Geronikaki A, Zoumpoulakis P (2010). Novel 4-thiazolidinone derivatives as potential antifungal and antibacterial drugs. *Bioorg. Med. Chem.*.

[44] Vicini P, Geronikaki A, Incerti M, Zani F, Dearden J, Hewitt M (2008). 2-Heteroarylimino-5-benzylidene-4-thiazolidinones analogues of 2-thiazolylimino-5-benzylidene-4-thiazolidinones with antimicrobial activity: synthesis and structure–activity relationship. *Bioorg. Med. Chem.*.

[45] Borenfreund E, Puerner J (1985). A simple quantitative procedure using monolayer cultures for cytotoxicity assays (HTD/NR-90). *J. Tissue Cult. Methods*.

[46] Fotakis G, Timbrell JA (2006). *In vitro* cytotoxicity assays: comparison of LDH, neutral red, MTT and protein assay in hepatoma cell lines following exposure to cadmium chloride. *Toxicol. Lett.*.

[47] GraphPad Software http://www.graphpad.com/.

[48] Prayong P, Barusrux S, Weerapreeyakul N (2008). Cytotoxic activity screening of some indigenous Thai plants. *Fitoterapia*.

[49] Kato N, Ikeda M, Mizutani T (1996). Replication of hepatitis C virus in cultured non-neoplastic human hepatocytes. *Jap. J. Cancer Res.*.

[50] El-Awady MK, Ismail SM, El-Sagheer M, Sabour YA, Amr KS, Zaki EA (1999). Assay for hepatitis C virus in peripheral blood mononuclear cells enhances sensitivity of diagnosis and monitoring of HCV-associated hepatitis. *Clin. Chim. Acta*.

[51] El-Nakkady SS, Roaiah HF, El-Serwy WS, Soliman AM, El-Moez SI, Abdel-Rahman AA (2012). Antitumor and antimicrobial activities of some hetero aromatic benzofurans derived from naturally occurring visnagin. *Acta Pol. Pharm.*.

[52] Inc. CCG (2009). *Molecular Operating Environment (MOE)*.

[53] RCSB Protein Data Bank http://www.rcsb.org/pdb/home/home.do.

[54] Howard S, Berdini V, Boulstridge JA (2009). Fragment-based discovery of the pyrazol-4-yl urea (AT9283), a multitargeted kinase inhibitor with potent aurora kinase activity. *J. Med. Chem.*.

[55] Flint TJ, Hirschberg E, MurryMR (1953). Comparative effects of chemotherapeutic agents on Brown-Pearce tumor and normal rabbit testis *in vitro*. *Proc. Soc. Exp. Biol. Med.*.

[56] Phillips MA (1928). CCCXVII.:the formation of 2-substituted benziminazoles. *J. Chem. Soc.*.

[57] Ozegowski W, Krebs D (1965). Aminosäureantagonisten. IV. Versuche zur Darstellung von [1-Methyl-5-bis-(β-chloräthyl)-amino-benzimidazolyl-(2)]-essigsäure. *Journal für Praktische Chemie*.

[58] Autenrieth W, Hinsberg O (1892). Ueber einige Derivate des o-Toluylendiamins. *Berichte der deutschen chemischen Gesellschaft*.

[59] Georgescu M (1892). Ueber einige Tetrahydroketochinoxaline. *Berichte der deutschen chemischen Gesellschaft*.

[60] Hinsberg O (1892). Ueber Methylhydrooxytoluchinoxalin und die Constitution der aus α-Oxysäuren und Orthodiaminen entstehenden Verbindungen. *Berichte der deutschen chemischen Gesellschaft*.

[61] Bistrzycki A, Przeworski G (1912). Die Konstitution der Verbindungen aus o-Diaminen und α-Oxysäuren. Acetylierung von Benzimidazolen. *Berichte der deutschen chemischen Gesellschaft*.

[62] Leng Z, Tao A, Xia Z, Gu X, Jin L, Wang R (1990). Synthesis of the derivatives of 2-acetylbenzimidazolyl thiosemicarbazone. *Zhongguo Yaoke Daxue Xuebao*.

[63] Kira MA, Abdel-Rahman MO, Gadalla KZ (1969). The vilsmeier-haack reaction – III Cyclization of hydrazones to pyrazoles. *Tetrahedron Lett.*.

[64] Bernard M, Hulley E, Molenda H, Stochla K, Wrzeciono U (1986). [Azoles. 17. Beta-(4-pyrazol)acrylic and propionic acids and their anti-inflammatory activity]. *Pharmazie*.

[65] Prasath R, Bhavana P, Ng SW, Tiekink ER (2011). 3-(4-Meth-oxy-phen-yl)-1-phenyl-1H-pyrazole-4-carbaldehyde. *Acta Crystallogr. Sect. Sect. E: Struct. Rep. Online*.

[66] Boyum A (1968). Isolation of mononuclear cells and granulocytes from human blood. Isolation of monuclear cells by one centrifugation, and of granulocytes by combining centrifugation and sedimentation at 1 g. *Scand. J. Clin. Lab. Invest. Suppl.*.

[67] Rodriguez-Antona C, Donato MT, Boobis A (2002). Cytochrome P450 expression in human hepatocytes and hepatoma cell lines: molecular mechanisms that determine lower expression in cultured cells. *Xenobiotica*.

[68] Hevener KE, Zhao W, Ball DM (2009). Validation of molecular docking programs for virtual screening against dihydropteroate synthase. *J. Chem. Inf. Model.*.

[69] Schrödinger L *PyMOL Molecular Graphics System*.

