# Supplementary material for: Dual inhibitors of hepatitis C virus and hepatocellular carcinoma: design, synthesis and docking studies
Source: Future Sci OA. 2017 Oct 25;4(1):FSO252. doi: 10.4155/fsoa-2017-0075 (PMC5729604; doi:10.4155/fsoa-2017-0075)
Supplement: Supplementary file 1 [file fsoa-04-252-s1.docx]

**Table 1: Results of the *in vitro* hemolytic assay**

| **Compound ID** | **% Hemolysis** | **Compound ID** | **% Hemolysis** |
| --- | --- | --- | --- |
| **5a** | 3.27 | **5q** | 3.49 |
| **5b** | 4.22 | **5r** | 1.87 |
| **5c** | 1.65 | **5s** | ND |
| **5d** | 1.05 | **5t** | ND |
| **5e** | 3.82 | **5u** | ND |
| **5f** | 4.59 | **5v** | 3.66 |
| **5g** | 2.03 | **5w** | 3.53 |
| **5h** | ND^*^ | **5x** | 8.74 |
| **5i** | 2.97 | **6a** | 11.24 |
| **5j** | 3.01 | **6b** | 3.08 |
| **5k** | 4.44 | **6c** | 4.02 |
| **5l** | 2.89 | **6d** | 1.31 |
| **5m** | 11.74 | **6e** | 4.51 |
| **5n** | 9.52 | **6f** | 1.01 |
| **5o** | 15.05 | **6g** | 2.65 |
| **5p** | 4.62 | **6h** | 7.56 |

***^*^ND: Not determined (Insoluble in test solvent)***

**Table 2: Results of the *in vitro* anticancer screening against human HCC cell line HepG2**

| **Compound ID** | **Anticancer (HepG2)** | **Safe doses**  **(100% viability of HepG2)** |
| --- | --- | --- |
|  | **IC_50_**  **µg/ml** | **LD_0_**  **µg/ml** |
| **5a** | 172.56 | 10.15 |
| **5b** | 119.61 | 23.02 |
| **5c** | 143.29 | 5.88 |
| **5d** | 1137.87 | 163.55 |
| **5e** | 317.71 | 31.12 |
| **5f** | **69.44** | 10.39 |
| **5g** | 192.51 | 66.72 |
| **5i** | 120.32 | 0.05 |
| **5j** | **63.81** | 5.08 |
| **5k** | 150.39 | 56.74 |
| **5l** | **82.08** | 5.11 |
| **5p** | **46.51** | 13.22 |
| **5q** | **35.72** | 23.12 |
| **5r** | **11.01** | 0.181 |
| **5v** | 250.13 | 30.89 |
| **5w** | 1099.98 | 92.40 |
| **6b** | 353.59 | 11.07 |
| **6c** | **76.82** | 3.40 |
| **6d** | **34.28** | 7.31 |
| **6e** | 110.48 | 16.75 |
| **6f** | 153.79 | 35.33 |
| **6g** | 247.60 | 18.62 |
| **5-FU** | 0.90 | ND |

Table 3: Results of the *in vitro* cytotoxicity assay against PBMCs

| **Compound ID** | **LD_50_** | | **LD_0_** | |
| --- | --- | --- | --- | --- |
|  | **µg/ml** | **µg/ml** | |  |
| **5a** | 13.99 | 11.17 | |  |
| **5b** | 92.27 | 5.83 | |  |
| **5c** | 139.19 | 32.45 | |  |
| **5d** | 160.43 | 11.43 | |  |
| **5e** | 117.12 | 4.26 | |  |
| **5f** | 259.71 | 5.83 | |  |
| **5g** | 39.01 | 8.88 | |  |
| **5i** | 25.35 | 7.60 | |  |
| **5j** | 134.00 | 9.90 | |  |
| **5k** | 319.64 | 33.29 | |  |
| **5l** | 46.89 | 8.33 | |  |
| **5p** | 120.46 | 12.67 | |  |
| **5q** | 1246.96 | 190.45 | |  |
| **5r** | 44.00 | 7.33 | |  |
| **5v** | 43.40 | 13.85 | |  |
| **5w** | 36.28 | 16.03 | |  |
| **6b** | 61.46 | 18.18 | |  |
| **6c** | 28.67 | 11.41 | |  |
| **6d** | 14.32 | 5.21 | |  |
| **6e** | 329.03 | 19.22 | |  |
| **6f** | 1369.42 | 253.34 | |  |
| **6g** | 136.76 | 14.41 | |  |


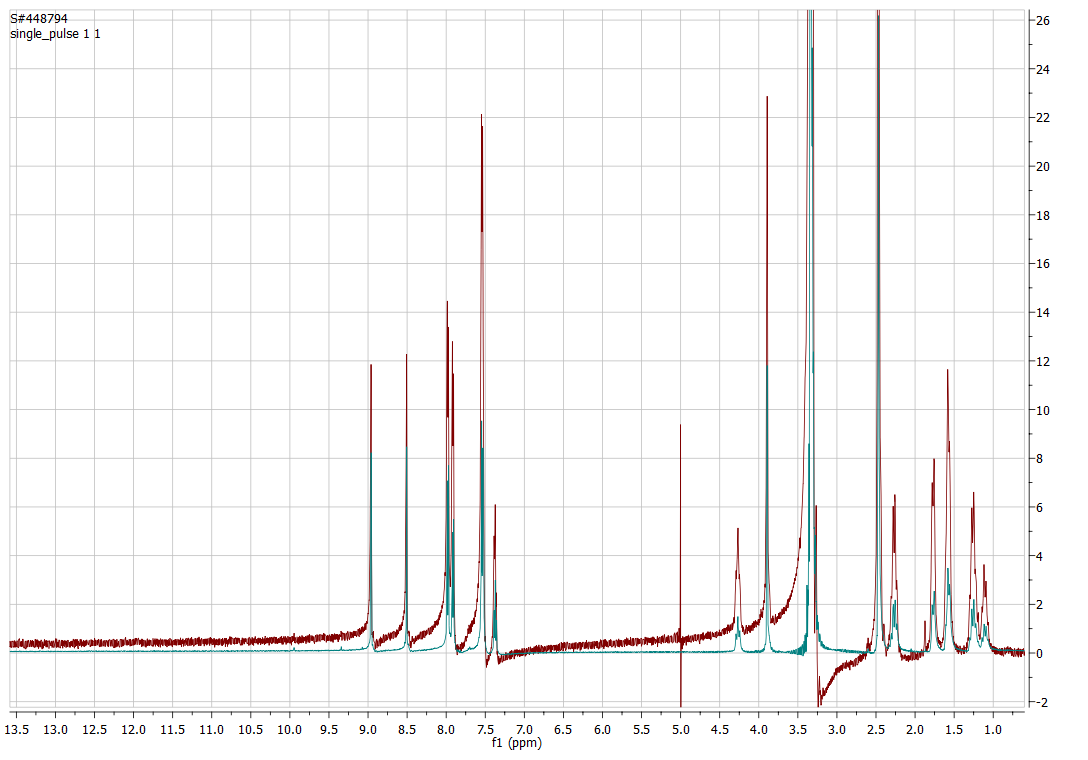


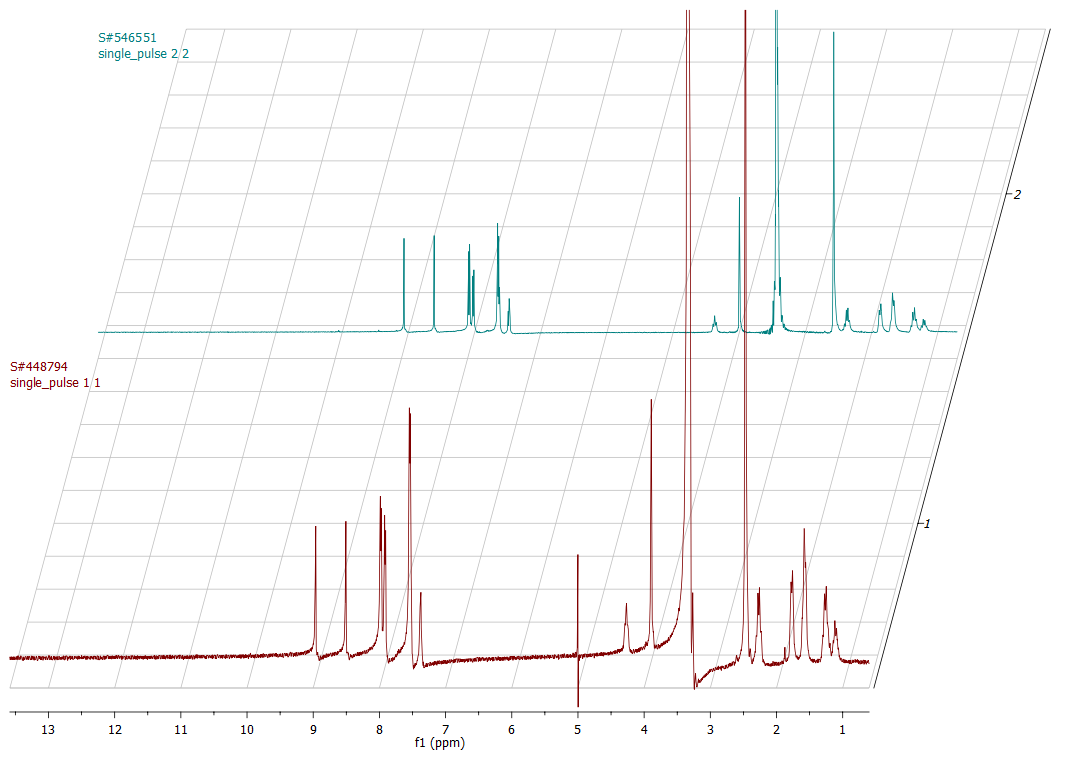


Figure1: Superimposition and stacking of the ^1^H-NMR spectra of the two unexpected products. The red chart corresponds to the product derived from benzimidazole derivative while the blue one corresponds to the product derived from benzofuran derivative

Figure 2: The proposed reaction mechanism for the formation of the unexpected compound (7)

**Part I, Spectra images**

Figure 1s: ^1^H-NMR spectrum of compound 2d


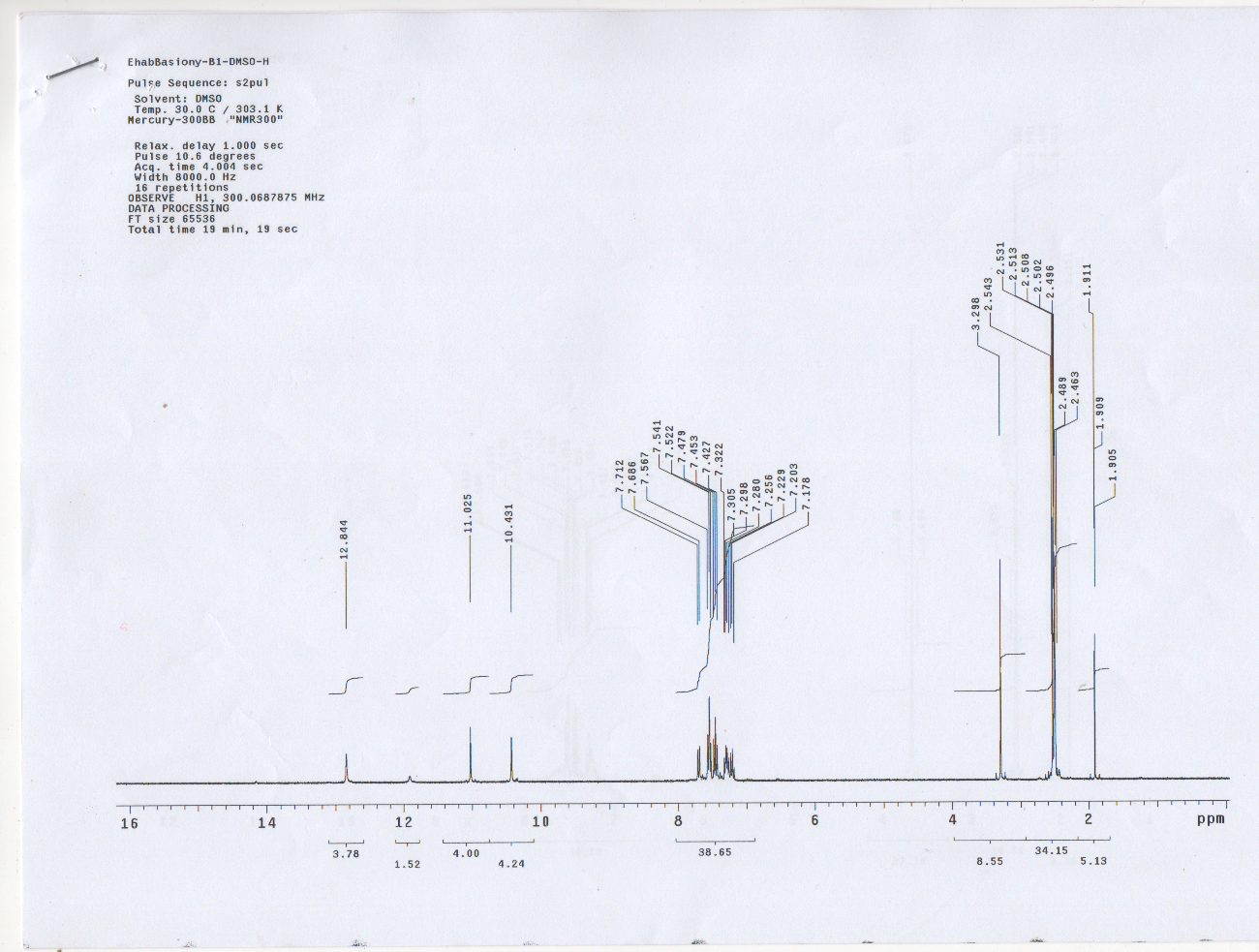


Figure 2s: ^1^H-NMR spectrum of compound 2e


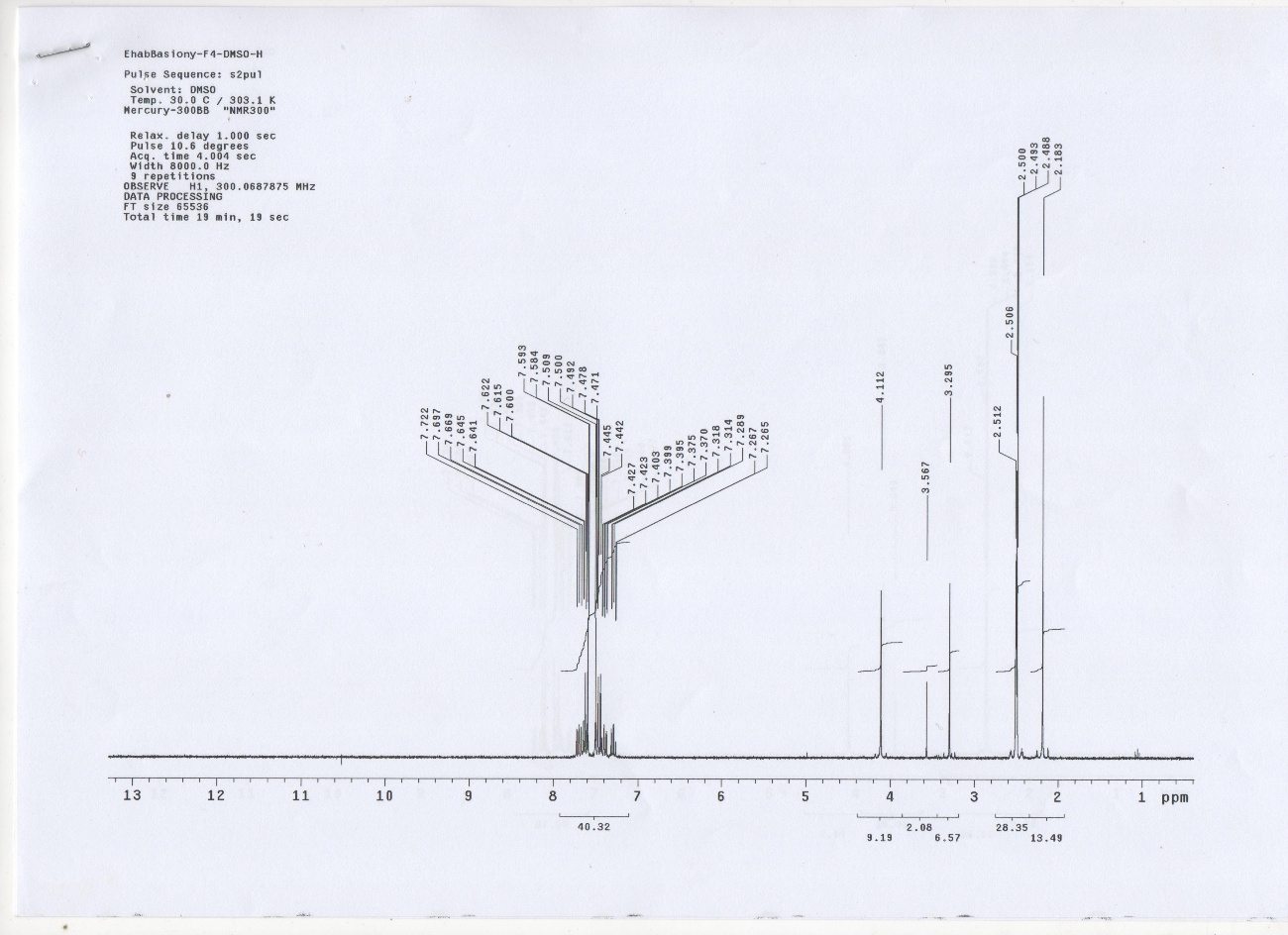


Figure 3s: ^1^H-NMR spectrum of compound 3b

Figure 4s: ^1^H-NMR spectrum of compound 3i


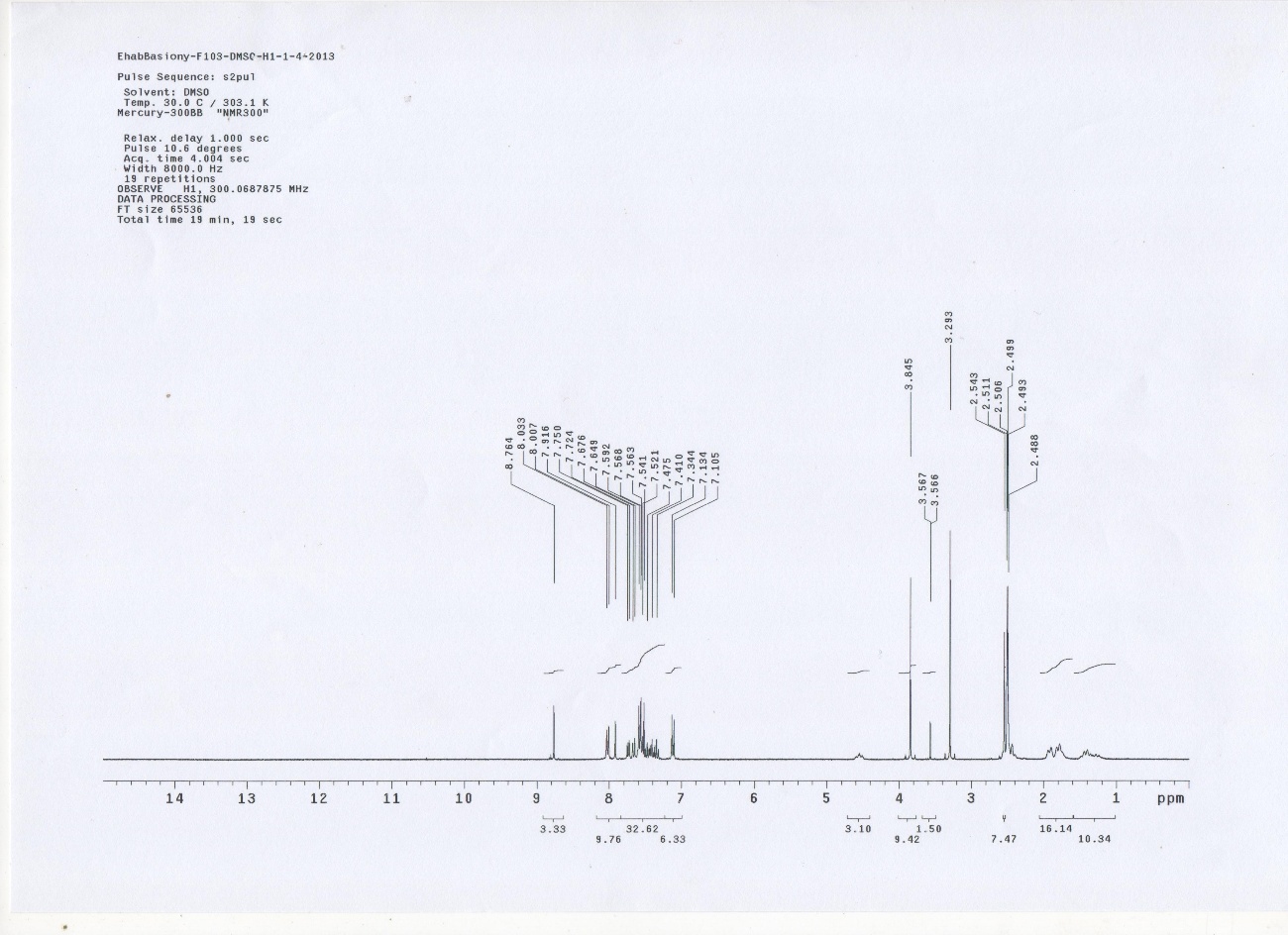


Figure 5s: ^1^H-NMR spectrum of compound 5l

Figure 6s: ^1^H-NMR spectrum of compound 5w


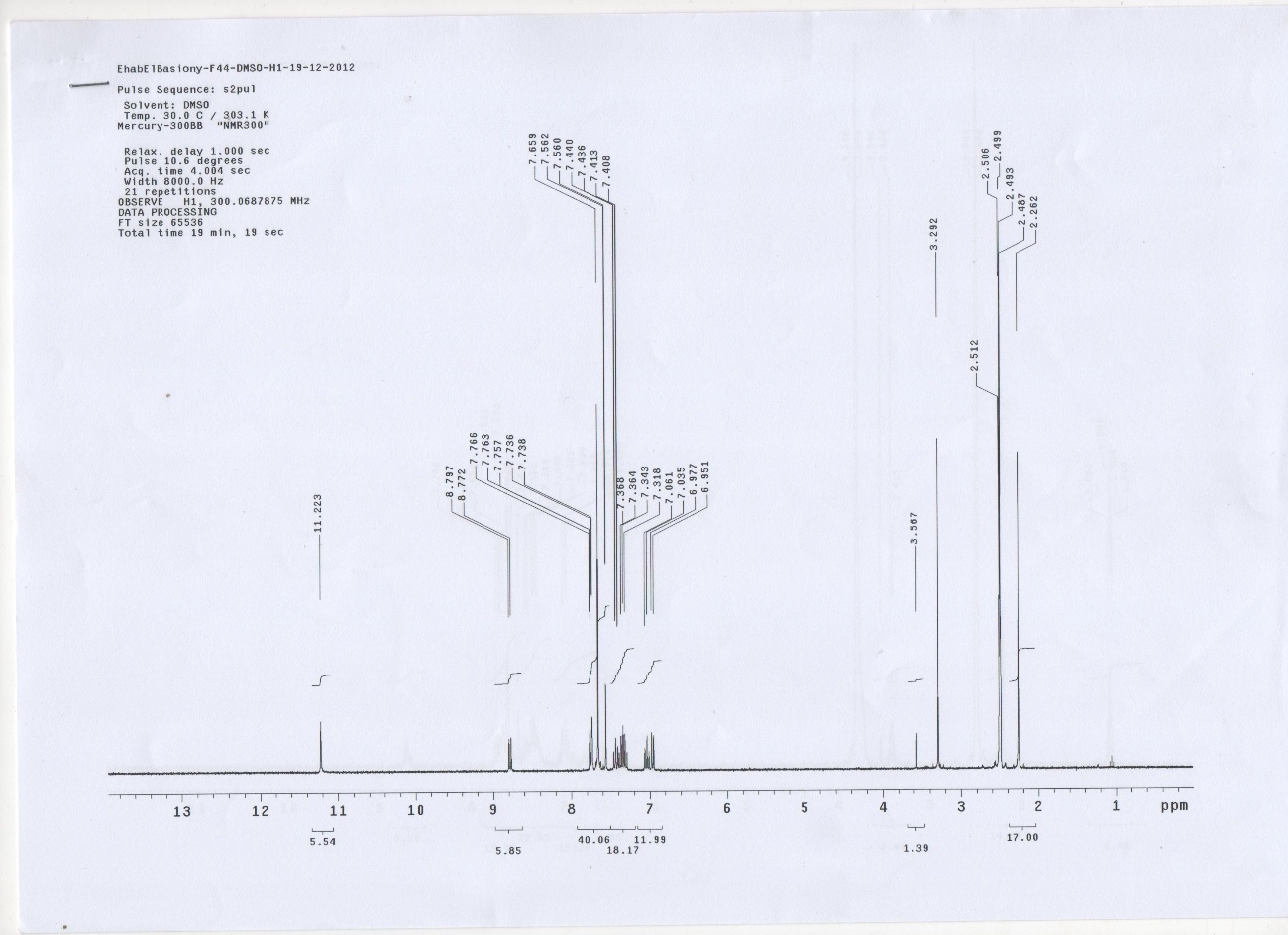


Figure 7s: ^1^H-NMR spectrum of compound 6b

Figure 8s: ^1^H-NMR spectrum of compound 6f


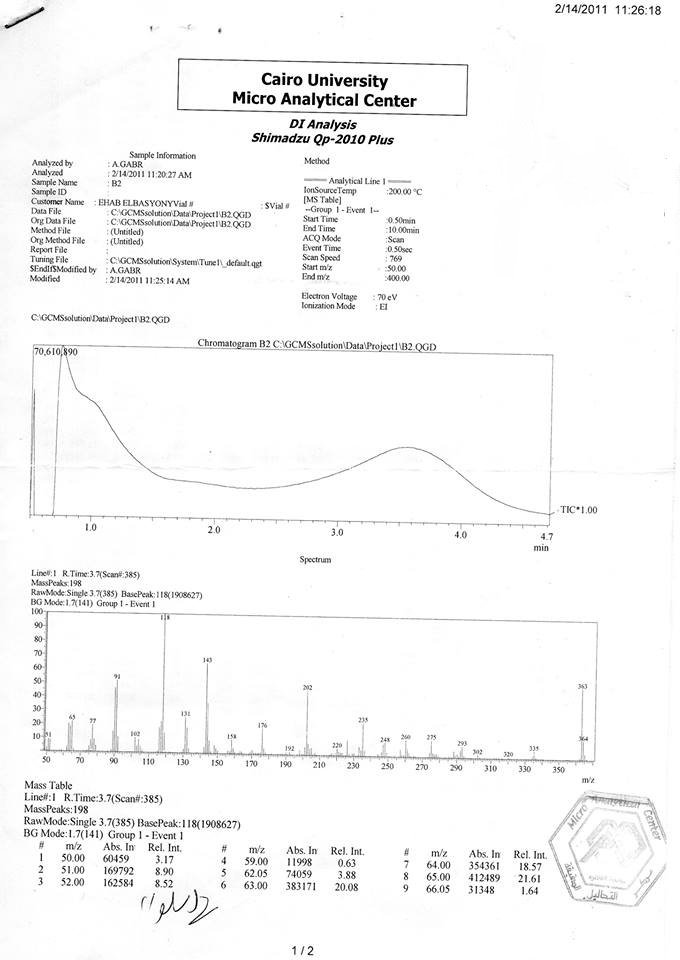


Figure 9s: Electron Impact Mass Spectrum of compound 3h


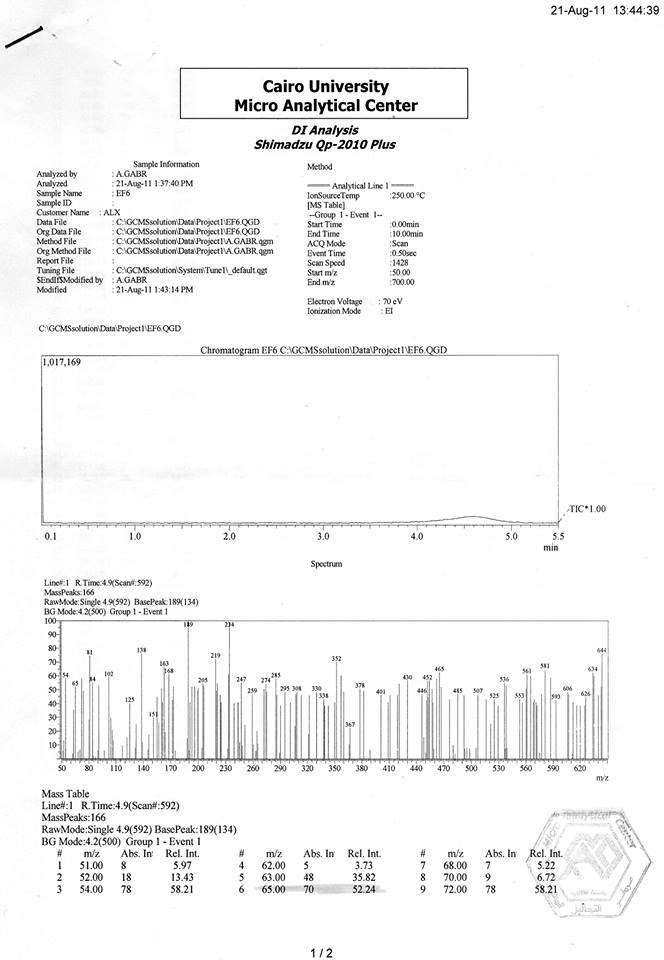


Figure 10s: Electron Impact Mass Spectrum of compound 5h


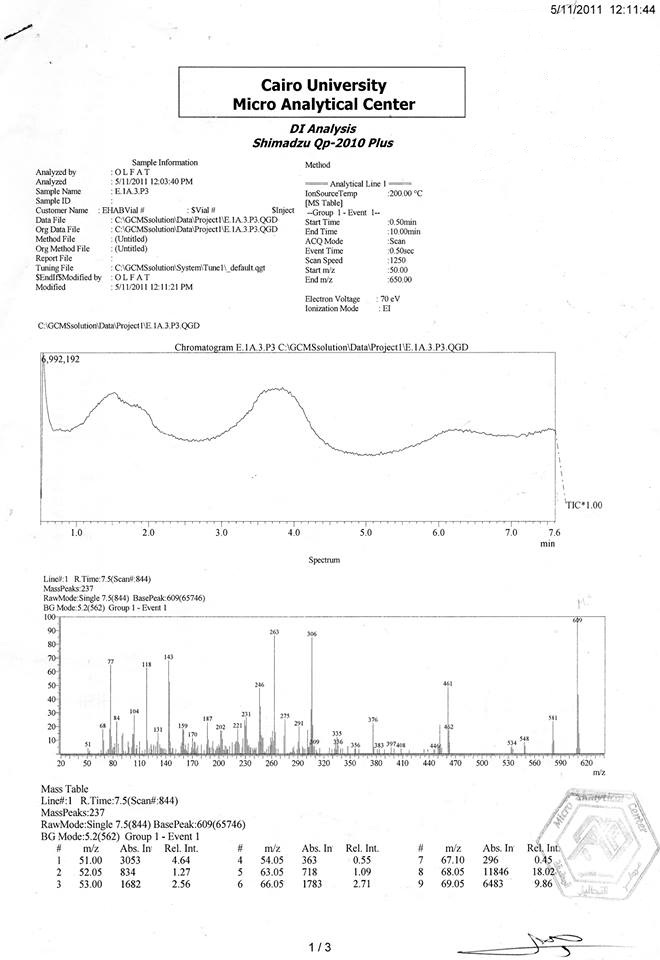


Figure 11s: Electron Impact Mass Spectrum of compound 5o


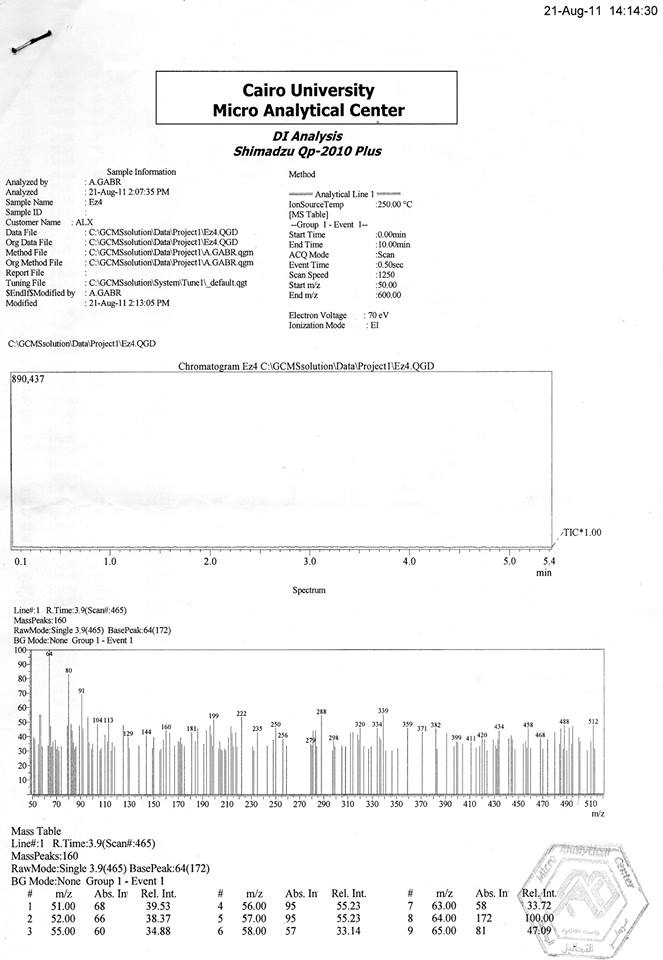


Figure 12s: Electron Impact Mass Spectrum of compound 6f

Figure 13s: IR spectrum of compound 2d

Figure 14s: IR spectrum of compound 2i

Figure 15s: IR spectrum of compound 3d

Figure 16s: IR spectrum of compound 3i

Figure 17s: IR spectrum of compound 5a

Figure 18s: IR spectrum of compound 5t

Figure 19s: IR spectrum of compound 5x

Figure 20s: IR spectrum of compound 6c

Figure 21s: IR spectrum of compound 6e

**Part II, Molecular Modeling**

Table 4: Docking scores (kcal/mol) of compounds 5a-x and 6a-h on VEGFR-2

| Compound ID | VEGFR-2 |
| --- | --- |
| Co-crystallized ligand and reference active compound (**Sunitinib**) | -7.14 |
| **5a** | **-7.23** |
| **5b** | **-7.21** |
| **5c** | **-7.32** |
| **5d** | -7.10 |
| **5e** | **-7.53** |
| **5f** | **-7.37** |
| **5g** | -7.03 |
| **5h** | **-7.20** |
| **5i** | **-7.26** |
| **5j** | **-7.48** |
| **5k** | **-7.31** |
| **5l** | **-8.45** |
| **5m** | -6.44 |
| **5n** | -6.89 |
| **5o** | **-7.36** |
| **5p** | **-7.67** |
| **5q** | **-7.74** |
| **5r** | **-7.37** |
| **5s** | -6.88 |
| **5t** | **-7.41** |
| **5u** | -6.95 |
| **5v** | **-7.34** |
| **5w** | **-7.19** |
| **5x** | **-7.50** |
| **6a** | -6.44 |
| **6b** | -6.89 |
| **6c** | **-7.36** |
| **6d** | **-7.67** |
| **6e** | **-7.20** |
| **6f** | **-7.49** |
| **6g** | **-7.47** |
| **6h** | **-7.83** |
